# Exopolysaccharide Anchoring Creates an Extreme Resistance to Sedimentation

**DOI:** 10.1128/JB.00023-21

**Published:** 2021-05-07

**Authors:** Nickolas G. Kessler, David M. Caraballo Delgado, Neel K. Shah, Jeff A. Dickinson, Sean D. Moore

**Affiliations:** a Burnett School of Biomedical Sciences, University of Central Florida, Orlando, Florida, USA; Université de Montréal

**Keywords:** *E. coli*, capsule, colanic acid, mucoid, sedimentation, *rcs*, RcsC, Lpp, IgaA, YjbF

## Abstract

By evolving strains of Escherichia coli that hyperresist sedimentation, we discovered an uncharacterized mechanism that bacteria can use to remain in suspension indefinitely without expending energy. This unusual phenotype was traced to the anchoring of long colanic acid polymers (CAP) that project from the cell surface. Although each characterized mutant activated this same mechanism, the genes responsible and the strengths of the phenotypes varied. Mutations in *rcsC*, *lpp*, *igaA*, or the *yjbEFGH* operon were sufficient to stimulate sedimentation resistance, while mutations altering the *cps* promoter, *cdgI*, or *yjbF* provided phenotypic enhancements. The sedimentation resistances changed in response to temperature, growth phase, and carbon source, and each mutant exhibited significantly reduced biofilm formation. We discovered that the degree of colony mucoidy exhibited by these mutants was not related to the degree of Rcs pathways activation or to the amount of CAP that was produced; rather, it was related to the fraction of CAP that was shed as a true exopolysaccharide. Therefore, these and other mutations that activate this phenotype are likely to be absent from genetic screens that relied on centrifugation to harvest bacteria. We also found that this anchored CAP form is not linked to lipopolysaccharide (LPS) cores and may not be attached to the outer membrane.

**IMPORTANCE** Bacteria can partition in aqueous environments between surface-dwelling, planktonic, sedimentary, and biofilm forms. Residence in each location provides an advantage depending on nutritional and environmental stresses, and a community of a single species is often observed to be distributed throughout two or more of these niches. Another adaptive strategy is to produce an extracellular capsule, which provides an environmental shield for the microbe and can allow escape from predators and immune systems. We discovered that bacteria can either shed or stably anchor capsules to dramatically alter their propensity to sediment. The degree to which the bacteria anchor their capsule is controlled by a stress sensing system, suggesting that anchoring may be used as an adaptive response to severe environmental challenges.

## Abstract (precis)

Bacteria can partition in aqueous environments between surface-dwelling, planktonic, sedimentary, and biofilm forms. Residence in each location provides an advantage depending on nutritional and environmental stresses, and a community of a single species is often observed to be distributed throughout two or more of these niches.

## INTRODUCTION

We observed that approximately one in 1,000 Escherichia coli cells remained suspended in their growth medium after a conventional centrifugal harvest (e.g., 5 min at 5,000 relative centrifugal force [RCF]). When the suspended cells were repropagated, the resulting cultures sedimented similarly, indicating that there was a nongenetic, physiological alteration that allowed a subpopulation to remain in suspension. Bacterial sedimentation is an important aspect of environmental science, biofouling, pathogen exposure, and water treatment, so we sought to identify this sedimentation resistance mechanism with the hope that it could be controlled or exploited. From this investigation, we discovered that E. coli can produce stably attached exopolysaccharides that substantially reduce its sedimentation. It was also discovered that the strength of this phenotype is governed, in large part, by the fraction of exopolysaccharide that remains attached to the cells.

Bacterial cell envelopes are highly dynamic, and their compositions and architectures are adjusted in response to environmental challenges, such as changes in temperature, hydration, or chemistry. In addition, perturbations to the production, transport, or quality of envelope macromolecules can invoke dedicated stress responses designed to compensate for the defects (1–3). One hallmark of envelope stress in enterobacteria is the production of an exopolysaccharide capsule, which is a loosely associated forest of sugar polymers that surround the cells. When grown on solid surfaces, capsule-producing colonies are mucoid, and some bacteria remain in the mucoid state in the absence of envelope stress (4–10). Although the chemical composition and presentation of stress capsules can vary temporally within a species or within a clonal population, their functional utility in protecting the cell is well established (2, 11–13). The mucoid phenotypes of capsule-producing colonies have served as a powerful visual tool that has been used to evaluate envelope health and to identify genes that are needed to build the envelope and to regulate envelope stress responses. It is from such screens that the *rcs* genes were discovered as regulators of capsule synthesis in response to envelope stress (9, 14, 15).

The architecture and stress response systems of the Gram-negative cell envelope have been studied extensively because of the envelope’s important role in maintaining cell health, and it is a target of many clinically important antibiotics. The outer leaflet of the outer membrane contains lipid A connected to a core oligosaccharide that serves as an anchor for a collection of polysaccharides that can be displayed on the cell surface, including the O-antigen of lipopolysaccharide (LPS) (12, 16–18). The outer membrane is itself anchored to the cell wall via Lpp, an abundant lipoprotein whose synthesis is regulated by a dedicated small RNA of the σ^E^ envelope stress sensing system (3, 19–23). The health of the outer membrane and the width of the periplasm are both sensed by physical associations between the outer membrane sensor RcsF, the inner membrane protein IgaA, the phospho-relay protein RcsD, and the hybrid sensor kinase RcsC (24–28). Interruptions to the production or localization of these factors activates RcsC, which transfers phosphate to RcsD and ultimately to the cytosolic response regulator RcsB to mediate an adaptive response (2, 14, 29). Either alone or in partnership with RcsA, phosphorylated RcsB controls the expression of several genes involved in envelope maintenance and motility (2). A notable target of activated RcsB in E. coli is the *cps* operon, which encodes proteins that produce the oligosaccharide colanic acid (6, 9, 12, 30). Colanic acid subunits can be linked together to form long chains before secretion to form a mucoid capsule. To distinguish these forms, we refer to the large, nondialyzable colanic acid polymers as “CAP.”

In this report, we describe the behavior of E. coli mutants that exhibit an extraordinary resistance to sedimentation. The primary genes that were mutated to induce this phenotype are *rcsC*, *lpp*, and *igaA*, which activated capsule production via RcsB in otherwise healthy cells. We observed that the degree of colony mucoidy exhibited by these mutants was not a gauge of the level of Rcs pathway activation or capsule production; rather, it was a reflection of the amount of CAP that was ultimately shed from the cells. These observations suggest that polymer shedding may be a regulated feature of capsule production depending on the nature or degree of an envelope stress. We also discovered that the anchored CAP responsible for sedimentation resistance is not connected to lipid A cores and that it may traverse the periplasm or be anchored to the cell wall. This finding inspires a new model for how polysaccharide chains may be used to stabilize cell envelopes.

## RESULTS

### The evolution of E. coli mutants that resist sedimentation.

**FIG 1:**
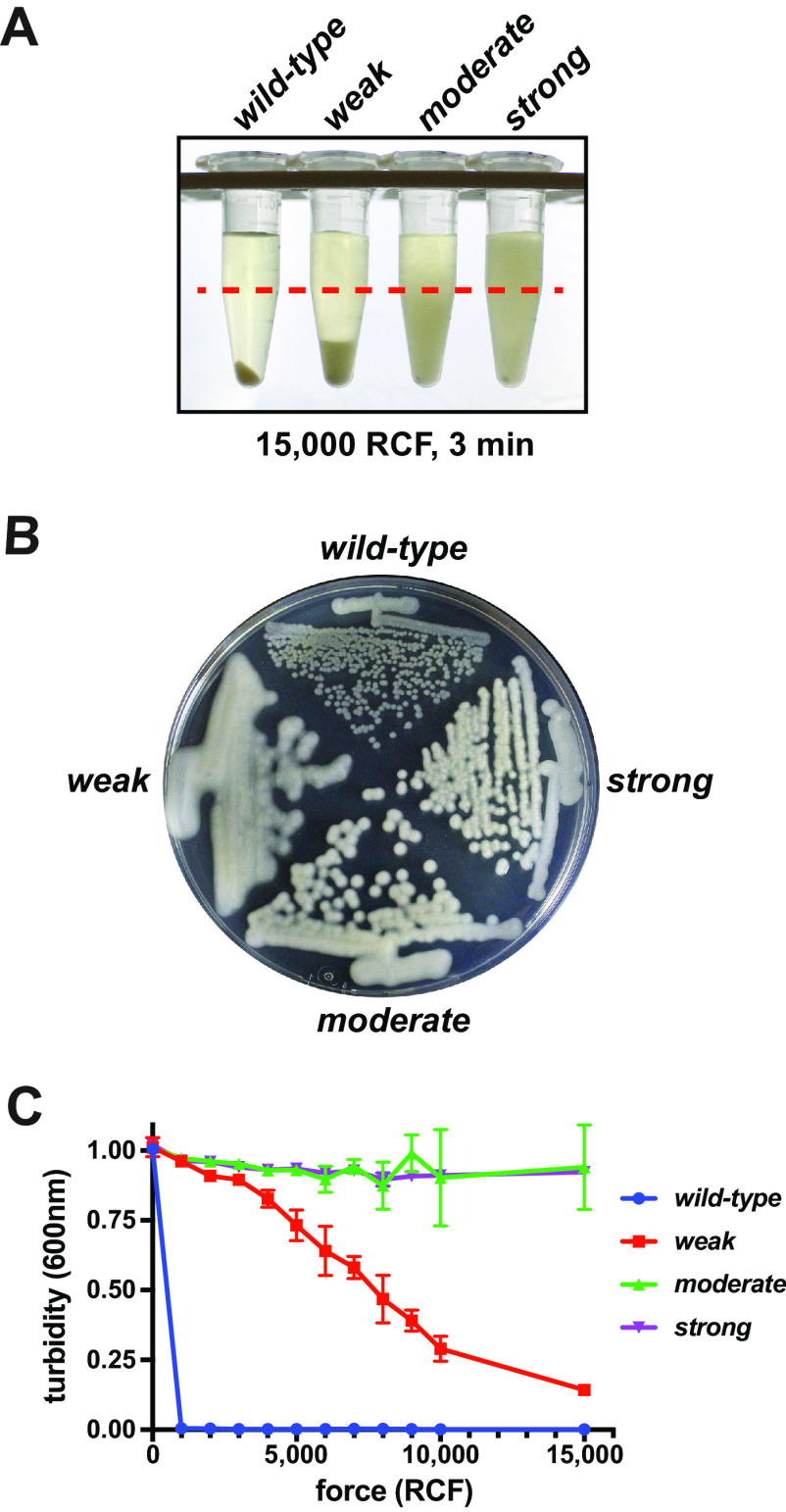
Three mutants that resist sedimentation. Serial passaging of culture supernatants selected for E. coli strains that resist sedimentation under high RCF. (A) Photograph of tubes containing 24-h cultures of either the wild-type parent or weak, moderate, or strong mutant that were centrifuged at 15,000 RCF for 3 min. The experimental sampling depth at the 0.5-ml mark is indicated with a red dashed line. (B) Solid agar plate (LB with 0.2% glycerol) of each strain grown overnight at 30°C. The mutant with the least sedimentation resistance exhibited the greatest mucoidy. (C) Sedimentation resistance as a function of applied force. First, 1-ml aliquots of 24-h cultures were centrifuged at the indicated forces for 3 min. A 100-μl aliquot of each supernatant was drawn from the 0.5-ml mark, and the turbidity was measured in a 96-well plate spectrophotometer at 600 nm. Error bars indicate standard deviations from three experimental replicates.

Strains that exhibit increased resistance to sedimentation were evolved by serially passaging samples of stationary-phase bacteria remaining in supernatants after centrifugation (see Fig. S1A in the supplemental material). The evolution cultures were grown for 24 h at 30°C before each evaluation. The strengths of sedimentation resistance were assessed at each stage by evaluating the distribution of cells in a 1-ml sample after a 10-min centrifugation at 3,300 RCF. Once a turbid supernatant was encountered in an evolution lineage (generally after 10 to 15 passages), an aliquot of the supernatant was plated on a solid agar medium, and a representative colony was selected. Each isolate was then retested to verify the phenotype and stored. From this collection, three mutants with differing phenotypic strengths (weak, moderate, and strong) were selected for additional study (Fig. 1A; Fig. S1B). Curiously, although each mutant exhibited differing degrees of colony mucoidy, the mucoidy and colony sizes were inversely proportional to their respective sedimentation resistances (Fig. 1B).

We characterized each mutant’s resistance to sedimentation as a function of applied force. After centrifugation of 1-ml culture samples at a given force, 100-μl samples were withdrawn from the supernatants and transferred to a 96-well plate for turbidity measurements (Fig. 1C). This experiment revealed that each strain was capable of sedimentation at high forces, but their responses to the applied forces varied according to their phenotypic strengths. The moderate and strong mutants had comparable supernatant turbidities under all tested forces, but visual inspection revealed that the cleared zones in the moderate mutant samples were larger than those in the strong mutant samples (e.g., Fig. 1A). During these higher-RCF tests, the suspended cells of each mutant formed nonuniform distributions, which caused greater variance in the replicate measurements. Nonetheless, each mutant exhibited a substantial resistance to sedimentation at forces well above that at which they were selectively evolved.

### Mutated genes associated with sedimentation resistance.

By identifying genome sequence variations relative to the parental strain (designated wild type), we found that the weak mutant contained a missense mutation in *rcsC* (L840R); the moderate mutant contained an in-frame deletion in *lpp* (ΔK26-A39), an IS*1* mobile element insertion in *yebE*, and an IS*1* insertion between *yjbE* and *yjbF*; the strong mutant contained a missense mutation in *igaA* (A564P), a complete deletion of *cdgI*, and IS*1* insertions in both the *cps* promoter and *yjbF* (Table 1). The IS*1* disruptions are detailed in Fig. S2 (31, 32). CdgI is a predicted inner membrane diguanylate cyclase (33–36), YebE is an inner membrane protein of unknown function (33), and YjbF is a predicted outer membrane lipoprotein whose gene is a member of the *yjbEFGH* operon (37, 38). This operon is regulated by the Rcs system and involved in the production of an exopolysaccharide that is distinct from colanic acid (39, 40). For presentation clarity, we maintain the weak, moderate, and strong strain designations of these strains until Discussion.

**TABLE 1:** Mutations associated with resistance to sedimentation

Mutant strain	Disrupted gene(s)	Reported function
Weak	*rcsC* L840R	Sensor kinase; regulates capsule production
Moderate	*lpp* ΔK26-A39	Tethers outer membrane to cell wall
*yebE*::IS*1*	Inner membrane protein
*yjbE-*IS*1-yjbF*	Rcs-regulated exopolysaccharide production
Strong	*IgaA* A564P	Rcs pathway regulator
*Pcps*::IS*1*	Promoter of the *cps* operon
Δ*cdgI-dgcJ′*	Inner membrane diguanylate cyclases
*yjbF*::IS*1*	Rcs-regulated exopolysaccharide production

**FIG 2:**
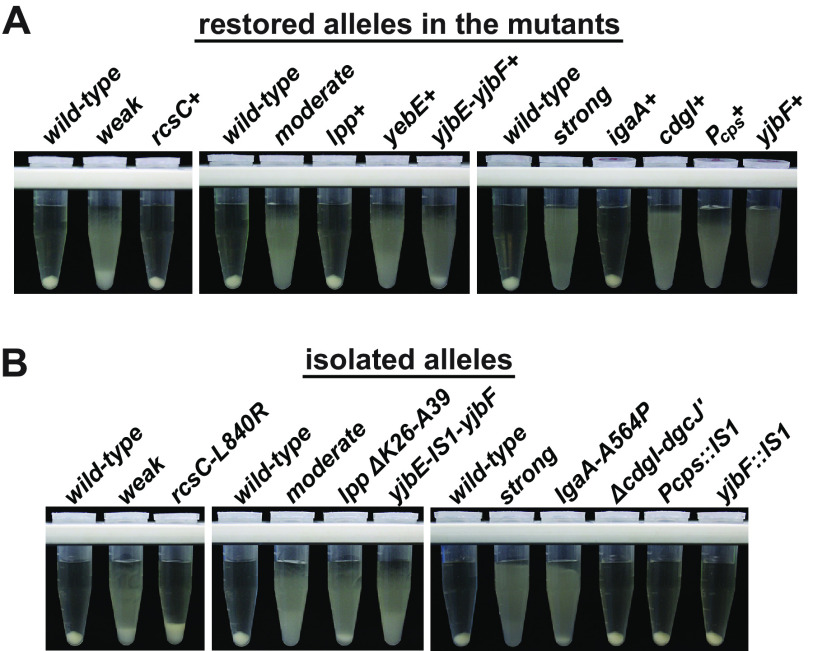
Phenotypes of restored and isolated mutations. Antibiotic resistance markers ∼5 to 10 kb away from the target loci in otherwise wild-type transduction donors were used to either restore each allele in the mutant strains or to relocate them into the naive parental strain using cotransduction. (A) Restoration of alleles in the mutant backgrounds. The restored alleles in each are denoted with a plus sign. The *rcsC*, *lpp*, and *igaA* restorations eliminated sedimentation resistance. (B) Isolation of the alleles. The isolated *rcsC-L840R* mutation was insufficient to fully reproduce the weak phenotype. We were unable to isolate the *yebE*::IS*1* allele using the same cotransduction marker that was used to restore it.

Using phage transductions of nearby drug resistance markers (41), we converted the mutant alleles back to wild type in each strain and subsequently scored the resulting phenotypes using centrifugation assays (Fig. 2A). Each of the targeted restorations reduced the sedimentation resistance phenotype of the host, with the exception of *yebE* in the moderate mutant. Restoring *rcsC*, *lpp*, or *igaA*, respectively, in the weak, moderate, and strong mutants completely eliminated their sedimentation resistance phenotypes. Therefore, the additional disruptions in the moderate and strong mutants appear to be auxiliary to the mutations in *lpp* and *igaA*.

We also transduced most of the mutations into a naive strain so that their influence on sedimentation could be evaluated independently (Fig. 2B). Isolation of the *rcsC-L840R* allele revealed that this mutation is sufficient to produce a mild phenotype; however, this phenotype was not as strong as that of the weak mutant, which suggests there is an unidentified mutation present in that mutant. The isolated *lpp* Δ*K26-A39* allele created a weaker phenotype than that of the moderate mutant and was similar to the phenotype of an ancestral strain that also carried *lpp* Δ*K26-A39* (Fig. S1B). Interestingly, the isolated IS*1* insertion between *yjbE* and *yjbF* caused an appreciable phenotype on its own. The isolated *igaA-A564P* allele from the strong strain provided a robust phenotype, but the other mutations isolated from this mutant did not cause sedimentation resistance.

For the four other sedimentation-resistant mutants whose genomes were not sequenced in this study, we Sanger sequenced PCR products derived from them that contained *lpp* or each of the Rcs signaling pathway genes—*rcsF*, *igaA*, *rcsC*, *rcsD*, *rcsB*, and the *cps* promoter along with their flanking regions. Two mutants from different generations of the moderate lineage had the same deletion in *lpp* (Fig. S1B), and the other two mutants had wild-type versions of all of these genes. We also evaluated the *cdgI* locus in these four strains using phage transductions that introduced large wild-type segments so that any contributions by regional genes could be simultaneously evaluated. There were no changes to the phenotypic strengths of these mutants when that locus was interrogated. Overall, while the genes primarily responsible for the sedimentation resistance in the weak, moderate, and strong mutants have been identified, it appears that additional genes can be involved in this phenotype.

### The mutations activate the Rcs-*cps* pathway via RcsB.

**FIG 3:**
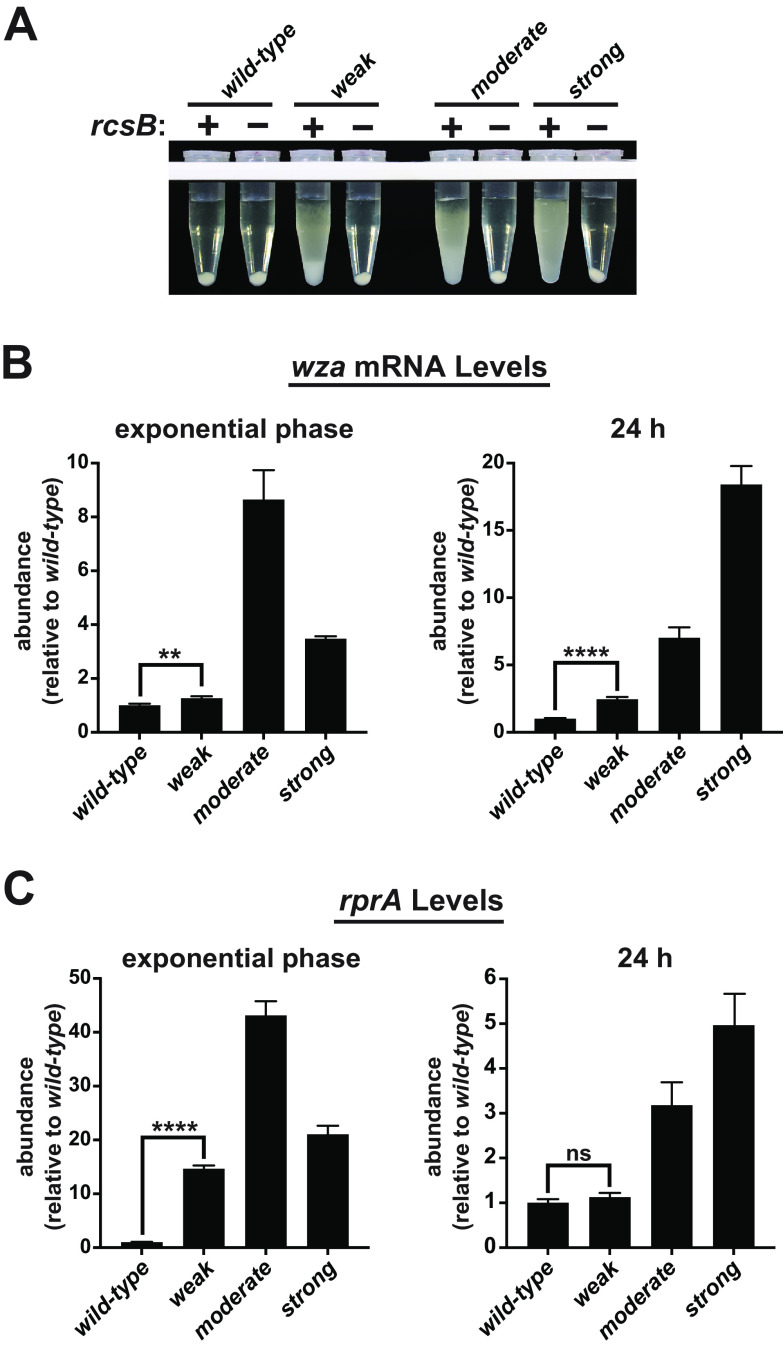
RcsB is necessary, and the Rcs system becomes activated. To evaluate the involvement of the Rcs pathway, the necessity of the RcsB response regulator and the expression of several target genes was measured. (A) Phage transductions were used to delete *rcsB* in each mutant, and the resulting sedimentation resistances were tested. The removal of this response regulator eliminated the phenotypes. (B) Total RNA was purified either from exponential-phase cultures (phenotypes off) or from 24-h cultures (phenotypes on) and converted to cDNA for quantification using real-time qPCR. Transcript levels are shown normalized to their levels in wild-type cultures. The qPCR signals from the target genes were first normalized to the amount of 16S rRNA within each sample, and then those values were normalized to wild-type levels between samples. The level of *wza* mRNA was elevated in all mutants under both culture conditions, but the relative abundance between the moderate and strong mutants shifted such that the mRNA levels trended with the phenotypic strengths at 24 h. (C) The abundance of the small regulatory RNA RprA followed a similar trend. The levels of these mRNAs were significantly higher than those of the wild type in all mutant samples except for the level of RprA in the moderate mutant at 24 h (*t* test *P* values < 0.01 to < 0.0001, not all indicated).

Literature precedents for the functions of the mutated genes, the *cps* promoter mutation, and the mucoid phenotypes were strong indicators that the Rcs envelope stress response pathway had become activated in these strains and that the *cps* operon had been induced to produce colanic acid via RcsB. To test this conclusion, we deleted *rcsB* from each mutant. Doing so completely eliminated the sedimentation resistance phenotypes and mucoidy, so the RcsB response regulator was necessary (Fig. 3A).

To directly evaluate activation of the Rcs system and induction of the *cps* operon, we purified total RNA from each strain to measure transcript levels, both from exponential-phase cultures (where the sedimentation phenotypes were largely absent) (Fig. S3A) and from 24-h cultures (where the phenotypes were pronounced). After preparing cDNA libraries, we used real-time quantitative PCR (qPCR) to establish the abundances of selected RNAs in the mutants and compared those to the amounts found in the parental strain. The 16S rRNA was used as an internal standard to allow comparisons between samples. The transcript levels of the first open reading frame (ORF) of the *cps* operon (*wza*) was significantly elevated in the mutants (Fig. 3B); in the exponential phase, the level was elevated by ∼1.3-fold in the weak mutant, ∼8.6-fold in the moderate mutant, and ∼3.5-fold in the strong mutant, whereas in the 24-h samples, these levels increased to ∼2.5-, ∼7.0-, and ∼18.4-fold, respectively. The *wcaD* and *wcaM* transcript levels (present in the middle and at the end of the *cps* operon, respectively) mirrored that of *wza* (not shown). We also measured the levels of the small regulatory RNA RprA because its promoter was used in prior studies as a reporter of Rcs activation (25, 27, 29). Although the relative expression levels of RprA were higher than those of *wza* in the exponential phase and lower in the stationary phase, they followed a similar trend among the mutants (Fig. 3C). We also measured the expression of *rcsA* and *yjbG* (which is downstream of and polycistronic with *yjbF*) (Fig. S3B). The levels of these transcripts were also elevated, with the exception that the level of *yjbG* mRNA was lower in the weak mutant in the exponential phase. Collectively, these observations indicate that the Rcs system had become activated in these mutants. Moreover, they reveal that the extent of activation correlated with the strengths of sedimentation resistance in the stationary phase and that they change as a function of growth stage.

### CAP is overproduced by the mutants.

**FIG 4:**
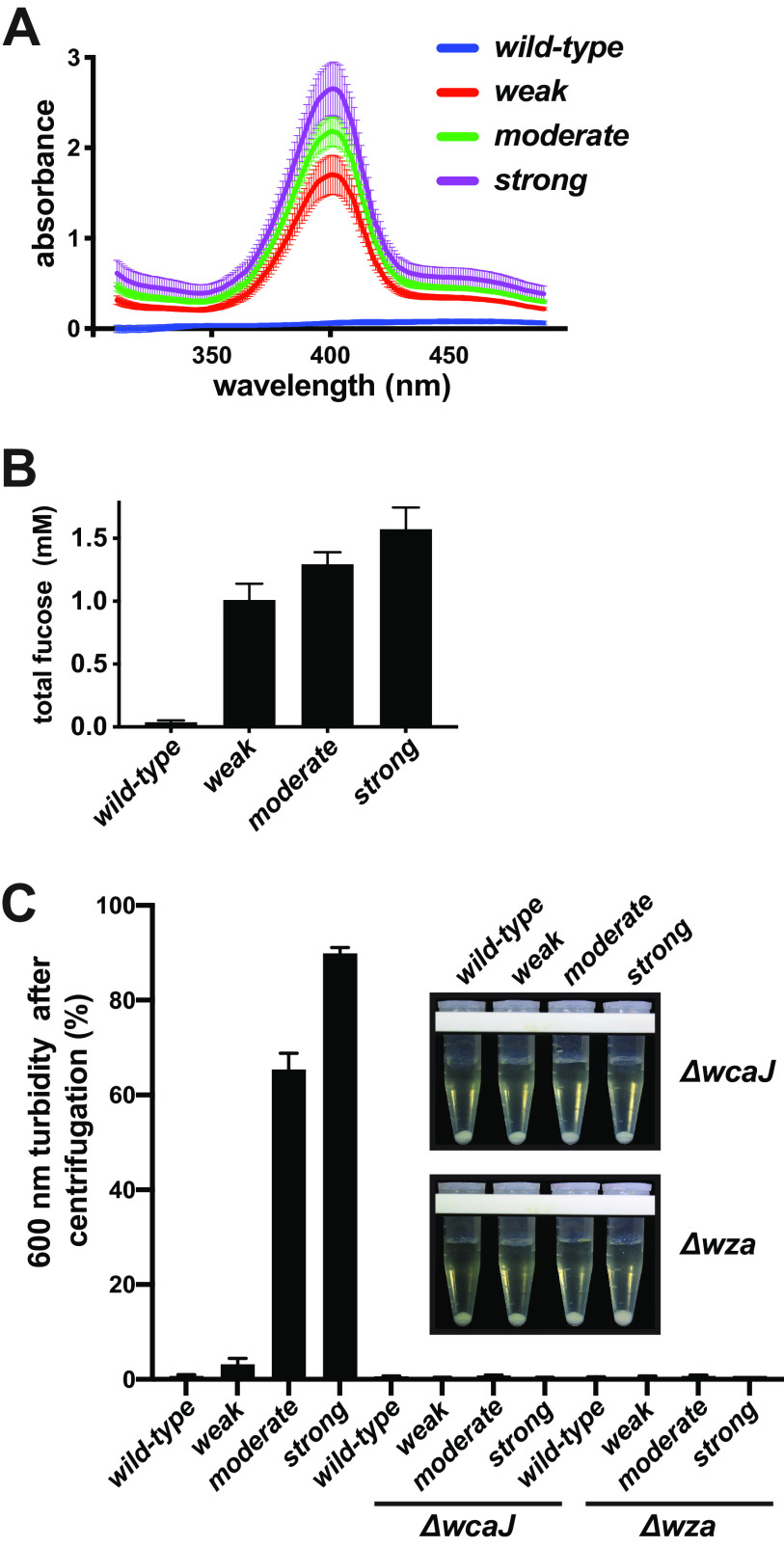
The mutants overproduce colanic acid polymer (CAP). Twenty-four-hour cultures were processed to extract nondialyzable exopolysaccharide, and the concentration of fucose was determined by comparison to a pure fucose standard curve. (A) Absorbance spectra of a fucose colorimetric assay on material purified from total cultures of wild-type and mutant strains. The diagnostic peak at 400 nm is indicative of methyl-pentose (fucose), which is a unique component of colanic acid. The curves show the average of three culture replicates (solid lines) and their standard deviations. Although there was a low signal at 400 nm for wild type that was greater than the blanks (depleted LB medium), the curve shapes suggest another chemical was causing absorbance, so the wild-type values are likely lower than those reported. (B) Using a pure fucose standard curve, the amounts of fucose produced by the cultures were quantified (wild-type, 36.8 ± 14.4 μM; weak, 1.01 ± 0.13 mM; medium, 1.29 ± 0.10 mM; strong, 1.57 ± 0.17 mM). (C) Genes encoding the beginning (*wcaJ*) and end (*wza*) of the CAP production pathway were deleted in the wild-type and mutant strains, and their sedimentation resistances were subsequently evaluated. After centrifugation for 10 min at 3,300 RCF, 100-μl aliquots were withdrawn from the 0.5-ml position, and their turbidities were compared to the uncentrifuged cultures and plotted as a percentage. The inset shows a representative set of each collection after centrifugation. Although most of the weak mutant cells migrated toward the bottom in these experiments, the amounts remaining at the sampling positions were significantly lower when *wcaJ* and *wza* were deleted (*P* = 0.0184 and 0.0204, respectively; *n* = 3). Deletion of *wcaJ* or *wza* in the wild-type background did not significantly alter the very small amounts of remaining turbidity.

The activation of the *cps* operon suggested that the mucoidy was a result of increased production of CAP (2). To test this idea, we prepared total exopolysaccharides from overnight cultures and then quantified the amount of fucose, which is a unique component of colanic acid polysaccharide (Fig. 4A) (4, 5, 42, 43). The amount of fucose present in wild-type cultures was ∼35 μM, whereas the amounts produced by the mutants ranged from ∼1 to 1.5 mM, approximately 35 times higher (Fig. 4B).

The observation that fucose was overproduced by the mutants suggested that CAP itself was responsible for the resistance to sedimentation; however, there are many Rcs-regulated genes and other polysaccharides produced by E. coli that may have contributed to this phenotype. To clarify the role of CAP in these mutants, we separately deleted the genes that encode the glucose-1-phosphate transferase (*wcaJ*) that initiates assembly of colanic acid repeats onto undecaprenyl-phosphate (Und-P) membrane anchors, as well as the gene encoding the outer membrane pores through which CAP is transported (*wza*) (44–47). When either gene was deleted, the mutants completely lost their sedimentation resistance phenotypes and mucoidy (Fig. 4C). Taking these data together, and with the caveat that the deletion of *wza* may have disrupted expression of the downstream *cps* operon, CAP production was necessary for the resistance to sedimentation. We additionally sought to determine if *cps* activation was sufficient by placing this operon under the control of a different promoter. Because of the operon’s large size, we attempted to replace the *cps* promoter in the chromosome using recombineering. Unfortunately, several attempts to generate two different P_lacZ_-*cps* fusions were unsuccessful, and the few recovered recombinants had disrupted *lacZ* promoters, suggesting this may be a lethal combination.

### Sedimentation resistance is a function of the fraction of anchored CAP.

The discovery that each mutant produced similar amounts of CAP was puzzling because they exhibited notably different sedimentation profiles and mucoidy. Several observations suggested that CAP had to be attached to the cells to cause sedimentation resistance and not that the media had been viscously conditioned or that the cells were connected to each other by some form of mesh. For example, mutant cells that were serially diluted more than a millionfold in fresh medium exhibited the same sedimentation resistances as their dense cultures (Fig. S4A). In addition, when wild-type cells were mixed into cultures of the mutants, the wild-type cells migrated and pelleted normally (Fig. S4B).

**FIG 5:**
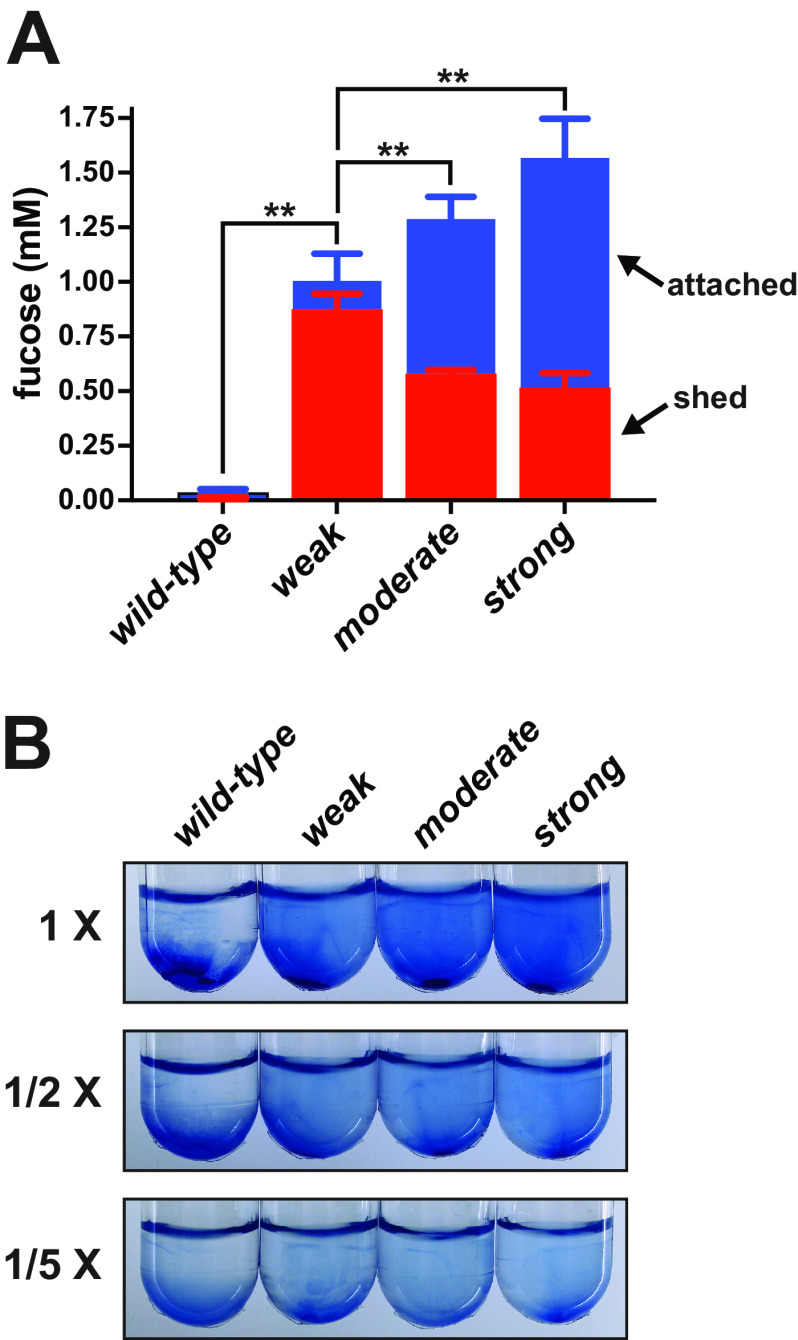
CAP shedding. The attachment and quality of CAP were evaluated. (A) Aliquots of the same cultures analyzed in Fig. 4A were centrifuged at 15,000 RCF for 30 min to create cleared supernatant zones sufficient for measuring cell-free fucose. The cell-free fucose was plotted on top of the total to illustrate the relative attached and shed fractions. The error bars represent the standard deviations of three experimental replicates. Asterisks represent *t* test *P* values of <0.01 that compared the shed percentages relative to the weak mutant to illustrate that this mutant hypersheds. (B) Exopolysaccharides were purified from each strain and incubated with blue synthetic microspheres to allow adsorption and then mixed 1:1 with fresh buffer, vortexed, and centrifuged at 3,200 RCF for 5 min to evaluate sedimentation resistance (labeled as 1×). Additional 2- and 5-fold dilutions of the adhesion mixtures in buffer did not eliminate the sedimentation resistances. Alternative centrifuge tubes were used for this assay to improve microsphere visualization.

We suspected that the differential phenotypes of the mutants were due to the amount of CAP that remained anchored to the cells. Attempts to separate the mutants from free colanic acid using filtration were unsuccessful because these CAP polymers did not pass through 0.45-μm filters. As an alternative approach, aliquots from the same cultures used to measure total colanic acid production (shown in Fig. 4) were centrifuged at a high RCF for 30 min to create cell-free zones that could be sampled near the surface. The colanic acid remaining in those cleared zones is considered to have been released from the cells (shed). Of the small amount of colanic acid produced by wild-type cultures, only ∼25% was shed (Fig. 5A). In contrast, the majority of the colanic acid produced by the weak mutant was released. The fractions of colanic acid shed by the moderate and strong mutants were significantly lower than that of the weak mutant.

It was also possible that the form of CAP produced by the weak mutant was less capable of producing a robust phenotype. To evaluate this idea, we purified CAP from cultures of each strain and adsorbed the material to synthetic microspheres. These mixtures were then centrifuged to evaluate sedimentation resistance. CAP produced by the weak mutant was as effective at retarding sedimentation as that produced by the moderate and strong mutants (Fig. 5B). Taken together with the striking differences in colony mucoidy, the variations in sedimentation resistance exhibited by the mutants were likely due to the fraction of CAP that remained attached to the cells.

### The mutations do not reduce envelope integrity.

**FIG 6:**
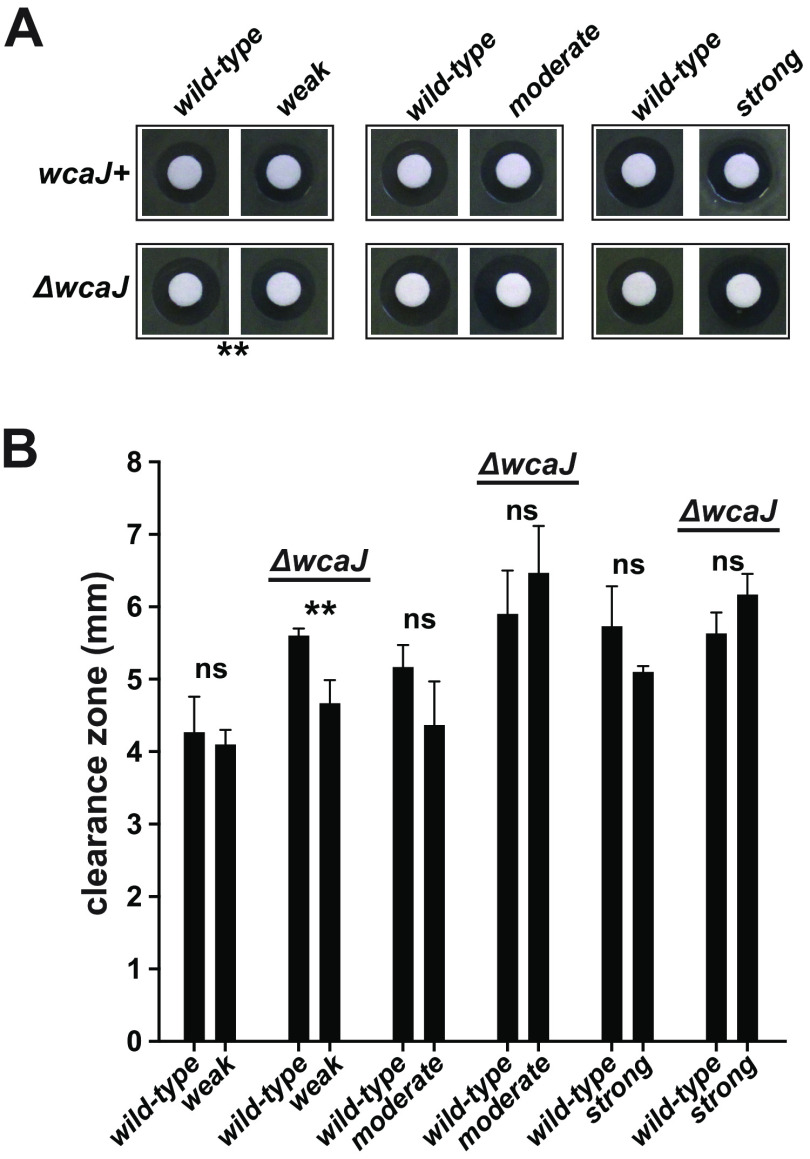
Envelope integrity in the absence of colanic acid. Resistance to SDS and EDTA was measured for each strain and its Δ*wcaJ* derivative. Bacteria were plated on LB-glycerol plates containing SDS, and then paper disks were placed on the inoculated areas and impregnated with an EDTA solution. After 16 h of growth, the lawns were imaged and the zones of clearance were measured. (A) Representative images of paper disks and clearance zones of each strain. (B) Clearance zones from three replicates were measured, averaged, and plotted. Although the clearance zones for wild type varied from plate to plate by over a millimeter, the clearance zones of each mutant were compared only to those of a wild-type counterpart on the same plate. Asterisks represent *t* test *P* values of <0.01; ns, not significant (*P* value of >0.05).

Did the alterations to these mutant proteins perturb the cell envelope such that an Rcs-mediated stress response became activated, or did those mutations invoke a response in the context of healthy envelopes? Having Δ*wcaJ* derivatives of each strain provided an opportunity to interrogate envelope integrities in the absence of colanic acid production. Each mutant, with or without *wcaJ*, exhibited similar growth rates, salt tolerances, antibiotic resistances, and persistent populations compared to the wild type, indicating that there were no overt defects in envelope permeability or stress responses (Fig. S5A to E, respectively) (48, 49). For a more aggressive interrogation, we measured each mutant’s resistance to EDTA in the presence of SDS using disk diffusion assays (Fig. 6) (20, 50, 51). The Δ*wcaJ* derivatives were not more sensitive to this chemical challenge; in fact, the weak mutant was slightly more resistant. Overall, colanic acid production is not a major defense against these environmental challenges, and the mutants do not have detectable envelope defects.

### CAP produced by these mutants is not attached to lipid A cores via WaaL.

**FIG 7:**
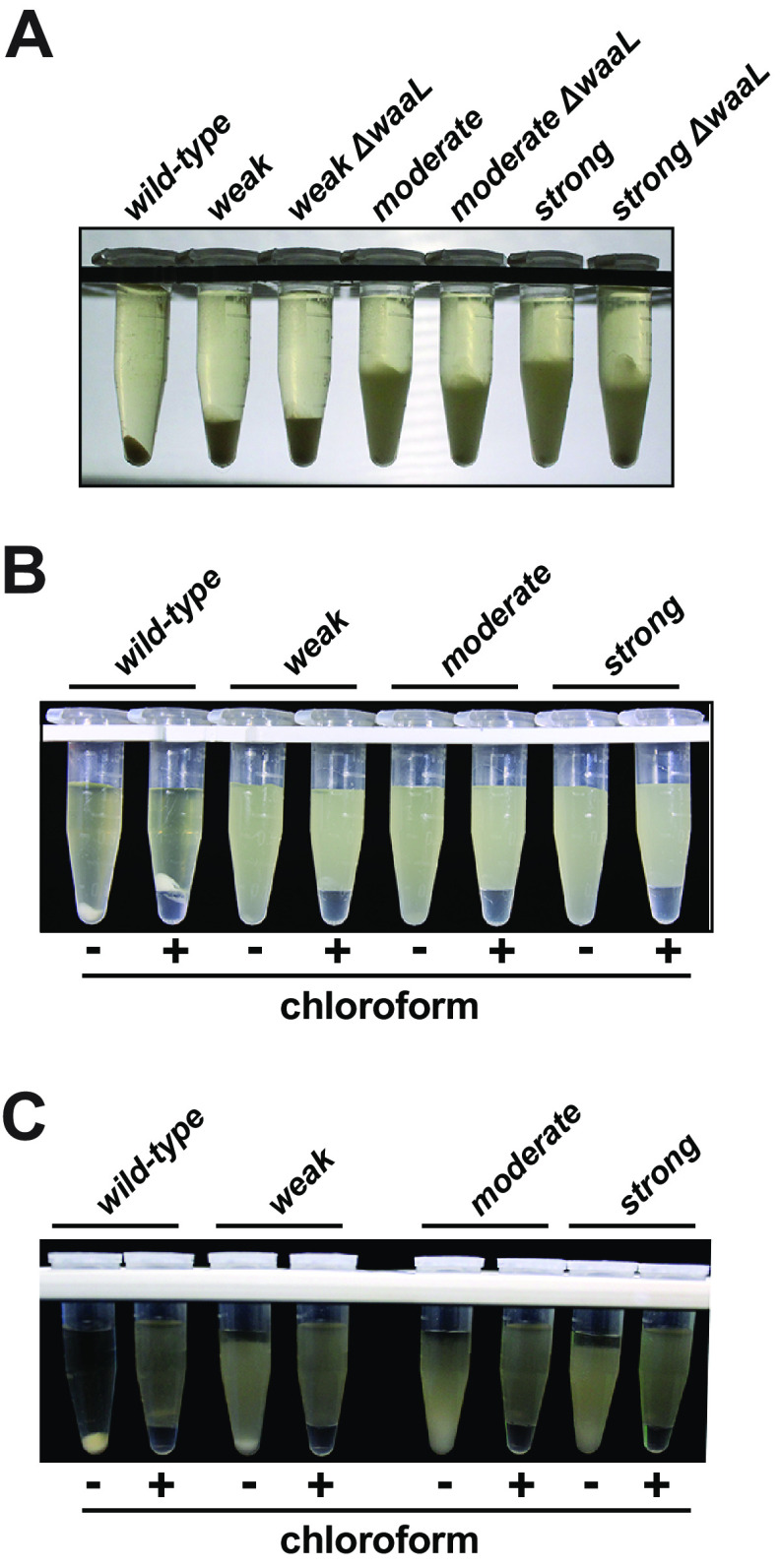
CAP is not attached to LPS cores *via* WaaL. The *waaL* gene was deleted from the mutants via transduction to characterize the resulting phenotypes. (A) Photograph of cultures centrifuged at 15,000 RCF for 30 min. The Δ*waaL* strains migrated slightly farther than their *waaL^+^* counterparts, but the overall sedimentation resistance remained pronounced. (B) Cultures of the wild-type and mutant strains were incubated with saturating chloroform for 30 min prior to centrifugation at 3,300 RCF for 3 min (to reduce migration of the weak mutant). There was no change in the sedimentation behaviors. (C) Cells expressing the phage T7 endolysin from a plasmid were treated with chloroform for 30 min prior to centrifugation at 3,300 RCF for 3 min. Each strain lysed, and the larger debris migrated to the chloroform interfaces.

On the periplasmic side of the inner membrane, several polysaccharides, including colanic acid, can be ligated to LPS core subunits prior to transport to the outer cell surface (12, 16, 17, 52–55). In E. coli, the only known glycosyltransferase capable of connecting polysaccharide substrates to the LPS core is WaaL, which was shown by another group to be necessary and sufficient for the attachment of colanic acid subunits (43). From these reports, we predicted that the CAP that caused sedimentation resistance was displayed from the outer membrane (OM) while being anchored by lipid A, similar to O-antigen. To our surprise, the Δ*waaL* mutants had indistinguishable sedimentation properties in lower-force centrifugation assays (not shown), and we detected only minor reductions in the phenotypes when they were centrifuged at high RCF for long periods of time (Fig. 7A). This interesting discovery raised the possibility that the cell-associated CAP were anchored to some alternative OM factor or that it was connected to a deeper envelope layer and displayed through the OM in a Wza-dependent manner.

Preliminary efforts to better characterize the connectivity and presentation of CAP were stifled by the aberrant sedimentation behavior of the cells and by the mutants having a potent resistance to extracellular lysozyme in the presence of EDTA (preventing, for example, the formation of spheroplasts). As an alternative, we employed a traditional method to extract hydrophobic compounds from the cells using chloroform, which solvates lipids but leaves most macromolecules and cell walls intact. When we treated the mutant cells with saturating amounts of chloroform for 30 min at room temperature, the resulting carcasses retained their sedimentation resistances, indicating that the CAP was still anchored (Fig. 7B). It was possible that the CAP layer was somehow resistant to chloroform diffusion and that the cell membranes had not been exposed sufficiently for disruption. To test this idea, we transformed the strains with a plasmid that expressed the phage T7 endolysin in the cytosol and subsequently evaluated the ability of chloroform to dissolve inner membranes and allow the endolysin to access the cell walls (56). This treatment caused thorough cell lysis in each transformed mutant strain, and cell debris sedimented to the chloroform interface similarly to that observed with the wild-type strain (Fig. 7C). Therefore, there was sufficient chloroform exposure through the CAP layers and cell envelopes to disrupt the inner membranes. Although the CAP produced by the mutants may have been attached to the outer membrane with some uncharacterized, chloroform-resistant anchor, taken this together with the *waaL* mutant data, we cautiously suggest that the anchored CAP responsible for the sedimentation resistance may have been attached to the cell wall or displayed through the cell wall while remaining attached to a large factor that cannot traverse the cell wall mesh, perhaps a CAP assembly factor.

### The sedimentation phenotypes are regulated and alter biofilm formation.

**FIG 8:**
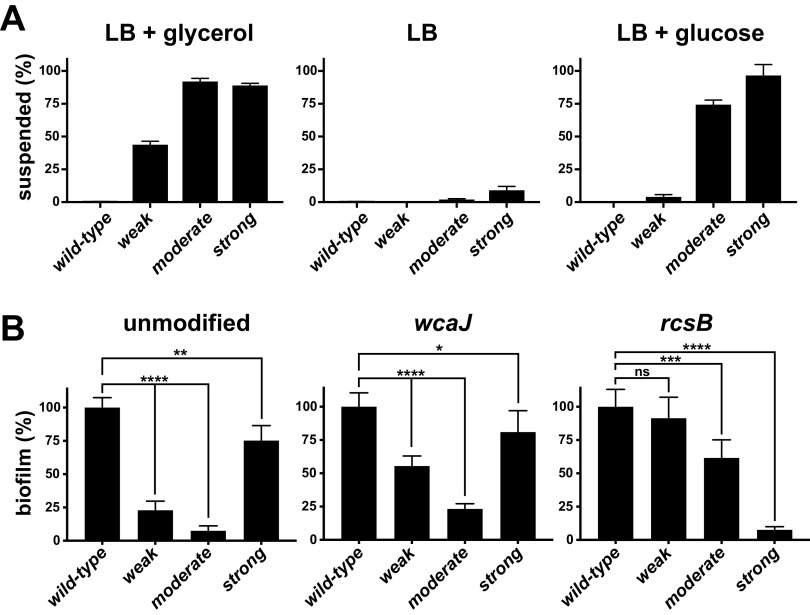
Regulation of sedimentation resistance and biofilm production. The mutants were characterized for changes in their sedimentation resistance when cultured with different available carbon sources and also for their propensities to form biofilms in static cultures. (A) The sedimentation resistances of cultures grown in LB supplemented with 0.2% glycerol (the selection medium), unsupplemented LB, and LB supplemented with 0.2% glucose were compared. These cultures were centrifuged at 10,000 RCF for 3 min to better reveal the weak phenotype. The turbidity remaining at the 0.5-ml position was plotted as a percentage of the starting turbidity for each culture. The supernatant turbidity of the wild-type cultures was essentially zero, so statistical comparisons were not performed. (B) Biofilm formation was measured in 96-well plates after 24 h of static growth at 30°C using crystal violet staining. The levels in each group (unmodified, *wcaJ* mutant, or *rcsB* mutant) were normalized to the amounts produced by the otherwise wild-type strains in each set assigned as 100%. Error bars represent the standard deviations of 6 independent cultures. Asterisks represent *t* test *P* values ranging from <0.05 (*) to <0.0001 (****); ns, not significant.

These sedimentation resistance phenotypes only became pronounced in the stationary phase, and the phenotypes were absent if the mutants were grown at 37 instead of 30°C. In addition, we discovered that the strengths of the phenotypes were dependent on the available carbon sources in the medium. For example, in nonsupplemented LB (stationary phase is carbon starved) (57), the phenotype was nearly absent from each mutant, while the phenotype of the weak mutant depended on glycerol (Fig. 8A). Although we have not yet established whether these phenotypic differences arose from changes in Rcs activation levels or anchoring efficiency, colonies formed on nonsupplemented LB are nonmucoid, suggesting an absence of CAP production.

We also evaluated the mutant phenotypes in a MOPS (morpholinepropanesulfonic acid)-buffered, rich, defined medium. Although the phenotypic strengths of the moderate and strong mutants were reduced compared to growth in LB, differential influences of glycerol and glucose remained, and the phenotype of the weak mutant was again enhanced by glycerol (Fig. S6A). A recent report revealed that E. coli prefers to consume certain amino acids for carbon instead of glucose (58), while other reports indicate that that E. coli can ferment glycerol under certain conditions (59, 60). Although our culturing was performed aerobically, which allows oxygen respiration, we were inspired to evaluate the mutant phenotypes in a minimal medium lacking amino acids to evaluate carbon source influences with fewer variables. For unknown reasons, neither the wild-type nor the mutant strains grew sufficiently in this medium with glycerol as a carbon source; however, with glucose, sedimentation resistances were largely eliminated except for that of the moderate mutant (Fig. S6B).

In an effort to tie the phenotypic regulation by the mutants to our original observation that ∼1/1,000 cells of the wild-type strain remain suspended, we reevaluated its sedimentation behavior when grown in the absence of supplemental glycerol. We observed no significant change in the fraction of cells that were resistant to sedimentation, so a glycerol supplement was not a requirement for that basal phenotype (Fig. S6C). Unexpectedly, preventing colanic acid production by deleting *wcaJ* from the parental strain did not reduce the proportion of sedimentation-resistant cells (Fig. S6C). Therefore, the anchoring of CAP is one mechanism to reduce sedimentation, but it is not the only mechanism.

The behaviors of the mutants indicated that CAP production prevented these cells from associating with each other when they were in the sedimentation-resistant state. We considered this property as a sort of “reverse biofilm” that promoted solo lifestyles. To evaluate the biofilm-formation capabilities of the unmodified mutants and their Δ*wcaJ* and Δ*rcsB* derivatives, we grew static cultures of each strain and quantified the amounts of polystyrene-adherent biofilm they generated. Each unmodified mutant formed significantly less biofilm than the wild-type cultures (Fig. 8B, left panel). Curiously, the biofilm amounts did not correlate with the strengths of sedimentation resistance. For example, the weak mutant produced more biofilm than the moderate mutant (∼25% versus ∼10%, respectively), while the strong mutant produced the most (∼75% of wild-type). When either *wcaJ* or *rcsB* was deleted in the wild-type background, the resulting strains each produced the same amount of biofilm within experimental error (not shown). In contrast, deletion of *wcaJ* approximately doubled the biofilm production in the weak and moderate mutant backgrounds (Fig. 8B, middle panel). Compared to the *wcaJ* deletion, the removal of *rcsB* largely restored biofilm production by the weak and moderate mutants; however, biofilm production by the strong mutant became substantially reduced (Fig. 8B, right panel). Because deleting *wcaJ* or *rcsB* eliminated the sedimentation resistance phenotype of these mutants, biofilm production in these assays was not a simple consequence of cells differentially settling onto the plastic surfaces. The CAP shedding and biofilm formation behaviors of these mutants suggests that they may be useful tools to investigate the molecular mechanisms underlying the transitions between planktonic and adherent behaviors.

## DISCUSSION

It is well known that the production of exopolysaccharides causes colony mucoidy that can make the handling of certain bacteria difficult (2, 10, 61, 62). A discovery we have presented is that mutations that strongly activate the Rcs system in E. coli appear to convert the production of CAP from a primarily secreted form to an anchored form that imparts an extreme resistance to sedimentation (13). Because the mutant cells migrated under high RCF, they were not buoyant neutral to the growth medium, so a likely explanation for their reduced sedimentation is that the anchored CAP had increased their hydrodynamic drag, akin to the pappus of wind-driven seeds. What this finding implies is that such mutants may have been missed in other genetic screens if their focus was on recovering highly mucoid colonies or if they involved centrifugation to harvest the cells. Further, our data suggest that CAP anchoring may be an adaptive response to combat severe envelope disruptions that hyperactivate the Rcs pathway.

Existing models of capsule production in E. coli describe the polymerization of colanic acid to form CAP in the periplasm and then either secretion or transfer of a few repeats to lipid A cores via WaaL before transport to the cell surface (6, 43, 63). Also, prior chemical analyses of colanic acid and its attachment site focused on the forms that were purified while being connected to lipid A (43, 55, 64, 65). It appears that the pool of colanic acid subunits connected to Und-P can be either used to decorate lipid A via WaaL or polymerized into CAP and displayed/secreted through Wza pores. We suspect the small reductions in overall CAP production in the Δ*waaL* strains resulted from the elimination of the fraction connected to lipid A. This model is reminiscent of the production of enterobacterial common antigen, which is destined to either become connected to lipid A (16, 66), transferred to phosphatidylglycerol, and displayed from the surface (67), or be polymerized into rings that remain in the periplasm (68, 69).

The RcsC-L840R mutation in the weak mutant is in the receiver domain that accepts phosphate from its histidine-kinase domain before delivery to RcsD. In the nuclear magnetic resonance (NMR) structure of the receiver domain, L840 makes direct contact with the carboxylate of the Asp residue that receives the signaling phosphate (D875) (70). Thus, we predict the flow of phosphate between RcsC and RcsD, in either direction, to be impacted by the positively charged arginine replacement. The elevation of *rprA* transcript levels in the exponential phase is indicative of a constitutive activation of the Rcs pathway; however, as with the other two mutants, the *rcsC-L840R* strain did not obtain appreciable resistance to sedimentation until the stationary phase. One possible explanation for this behavior is that an RcsD phosphatase activity that resets RcsB may be linked to the transfer of phosphate back onto RcsC, such that RcsB-P overaccumulated as the pool of RcsB aged. A similar model has been proposed by others to explain the behavior of a mutation structurally adjacent to Asp875 (A904V, *rcsC137*) in E. coli and two mutations near the corresponding Asp in the RcsC of Salmonella enterica, each of which leads to constitutive activation of the Rcs pathway (9, 25, 70, 71).

An alternative mechanism to explain the disconnect between Rcs activation levels and sedimentation resistance in the exponential phase is that some factor(s) required for CAP production or anchoring was differentially expressed. In either case, although the level of Rcs pathway activation in the weak mutant was notably lower than the other two, this mutant produced abundant CAP and was hypermucoid, again suggesting that phenotypic descriptions of mucoidy in other E. coli mutants, or even other bacteria, may not accurately reflect the degree of Rcs pathway activation or capsule production.

The spacing between the inner and outer membranes is important because there are physical interactions between stress sensors that report envelope structural defects to the cytosol (2, 23, 27, 28, 72). The *lpp* ΔK26-A39 allele found in the moderate mutant likely brings the outer membrane closer to the cell wall, which may cause misalignment of stress sensors or cause the exclusion of some OM proteins with large periplasmic domains. Another group recovered an in-frame deletion in *lpp* as a suppressor of the lethality imposed by the inability to produce phosphatidylglycerol phosphate (*lpp* Δ*L37-A57*) (73, 74). Interestingly, whereas full-length Lpp normally partitions between an essential cell wall-linked pool and an unlinked OM pool of unknown function (75), the ΔL37-A57 version resides almost exclusively as a wall-linked form (20). From these observations, we predict that the partitioning of the ΔK26-A39 Lpp in the moderate mutant is likely to be distorted as well. We also predict that the synthesis of these truncated Lpp mutants remains under the control of the σ^E^ envelope stress response pathway because the binding site for the small regulatory RNA MicL in these *lpp* transcripts is preserved (21, 76).

The *igaA-A564P* mutation was primarily responsible for the potent sedimentation resistance in the strong mutant, while the disruption of *yjbF* appeared to be independently additive. A mutated *igaA* was previously identified during a screen for *Salmonella* mutants that overgrow within fibroblasts, and that *igaA* mutation caused overproduction of exopolysaccharide (77). When protein folding is disrupted in the outer membrane, it is believed that RcsF is freed to associate with IgaA, which then removes the inhibition of RcsC signaling (26). Thus, defects in IgaA can lead to an inability to sense RcsF signaling and an overactivation of the Rcs pathway (26, 33, 72). A recent report demonstrated direct interactions between RcsD and IgaA, and it appears that a primary role of IgaA is to inhibit signaling from RcsC through RcsD (29). Therefore, it seems likely that the A564P mutation may weaken its association with either RcsF or RcsD because this mutation resides in the large periplasmic domain of IgaA (78).

Although the phenotypic impact of the *cdgI* deletion was relatively minor, it is predicted to be an inner membrane diguanylate cyclase and worth considering from a biofilm perspective (33, 36, 79). Cyclic di-GMP is a second messenger that triggers biofilm formation and an exit from the planktonic state, so it is not surprising that reducing the production of this molecule could help to drive an “antibiofilm” mode (35, 80). Countering this model, it is reported that deletion of *cdgI* dramatically increases early biofilm formation (34). In support of this observation, we found that the strong mutant produced more biofilm than the other mutants in our assay, despite having a robust sedimentation resistance and a high level of CAP production.

Although a detailed study of biofilm formation and motility was not in the scope of this investigation, we suspect that some of the disparities in the literature, especially with respect to the involvement of exopolysaccharides during biofilm formation, arise from differences in the form of colanic acid that was produced (and sedimentation/surface contact) and whether its production was coupled to the activation of other pathways necessary for biofilm development, such as the curli genes (81). It is worth noting that activated RcsB represses the curli-related *csg* operons (82), which can explain the observed reductions in biofilm formation by our mutants. Also of note, it is reported that a strain that cannot produce colanic acid (via a deletion of the *cps* operon, including *wcaJ*) causes a reduction in biofilm formation (83), which we did not observe in our wild-type background. We have not yet investigated whether this disparity stems from a culturing difference or the notably different biofilm assays used.

Aside from the IS*1* insertion between the *yjbE* and *yjbF* ORFs, the other mutations identified in the moderate and strong mutants produced no detectable phenotypes on their own. The IS*1* insertion upstream of *yjbF* is intriguing because the *yjbEFGH* operon is itself regulated by the Rcs system (39, 40), and we found that the *yjbG* message was elevated in all of the mutants in the stationary phase in a manner that mirrored the expression of the other Rcs targets we measured. In a preliminary characterization of a strain with an isolated *yjbE-IS1-yjbF* allele, we found that this mutant overproduced CAP in the stationary phase, which is consistent with the observed sedimentation resistance (not shown). Although, we have not yet measured transcript levels in this strain, we infer that the IS*1* insertion upstream of *yjbF* apparently causes the activation of an Rcs response, at least in terms of inducing the *cps* operon. Further characterization of the interplay between these two systems will be required to reveal a mechanistic link, but one possibility is that this mutation somehow perturbs the OM.

One unresolved question is how the production of a polysaccharide physically protects Gram-negative cells. An early report of colanic acid capsules enabling resistance to desiccation was later integrated into a model that included protection from osmotic shock (84, 85). It is clearly established that the Rcs pathway becomes activated as a response to such challenges, but it is not clear how the secretion of a hydrophilic sugar polymer would counter the effects of an external hydrophilic sugar such as sucrose (85–87). Moreover, colanic acid capsules do not appear to substantially alter the diffusion or accessibility of small molecules, nutrients, or antibiotics. Creating a network of *trans*-periplasm polysaccharide chains may help to mechanically stabilize or restore envelopes that have had the inner membrane pulled away from the cell wall. In addition, dehydration stress responses that anchor exopolysaccharides may enable bacteria to remain hydrated in suspension rather than associating with drying surfaces. A more detailed study of the suspension behaviors under different stresses and establishing the mechanism of CAP anchoring will be required to interrogate these models.

## MATERIALS AND METHODS

### Strains.

The parental strain designated as wild-type was an in-house Δ*rna*::*FRT* derivative of BW30270 (a prototrophic, *fnr^+^* *rph^+^* MG1655 strain; CGSC 7925) that was obtained from the Yale Coli Genetic Stock Center. The more common research strains MG1655 (*fnr*^+^ *rph-1*; CGSC 6300) and W3110 [IN(*rrnD-rrnE*)1 *rph-1*; CGSC 4474] were also obtained from that stock center (88). Although W3110 was derived from MG1655, it has substantial genetic differences that arose from extensive laboratory cultivation (89). DH5α [*endA1 glnV44 thi-1 recA1 relA1 gyrA96 deoR nupG purB20 ϕ80dlacZ*ΔM15 Δ(*lacZYA-argF*)*U169 hsdR17*] was obtained from New England Biolabs (Ipswitch, MA).

The following P1 transduction donor strains were generated in a previous study and were obtained from the Yale stock center (90): Δ*wcaJ*::*kan* (CGSC 9669) and Δ*wza*::*kan* (CGSC 9682) were used to block CAP production and secretion, respectively; Δ*waaL*(*rfaL*)::*kan* (CGSC 10649) was used to delete the O-antigen ligase; Δ*ydhC*::*kan* (CGSC 9406) and Δ*ydiB*::*kan* (CGSC 9425) were used to restore/relocate the *lpp* locus; Δ*ansA*::*kan* (CGSC 9476) and Δ*yeaX*::*kan* (CGSC 9501) were used to restore/relocate the *cdgI*(*yeaI*) locus; Δ*php*::*kan* (CGSC 10495) and Δ*gntX*::*kan* (CGSC 11520) were used to restore/relocate the *igaA* locus.

The following additional mutants were generated for this study using recombineering (91): *yfaP*::*tet* was used to restore/relocate the *rcsC* locus; *lamB*::*kan* was used for the *yjbF* locus, *alkA*::*tet* was used for the *cps* promoter, and *yebZ*::*kan* was used for the *yebE* locus. PCR products were stitched to create *tet*-*P_lacZ_* fusions that were used in attempts to place the *cps* operon under the control of the *lac* promoter, with and without the *P_cps_* JUMPstart element (63, 92). Recombineered loci were transduced to naive strains prior to generating P1 donor lysates, and all modifications and transductants were confirmed using combinations of diagnostic PCRs and Sanger sequencing.

### Evolution of sedimentation resistance.

For each round of selection, cultures were grown to stationary phase (24 h at 30°C) in 10 ml of Lennox lysogeny broth (LB; 0.5% NaCl) supplemented with 0.2% glycerol. Then, 1-ml culture samples were centrifuged at 3,300 RCF for 10 min in microcentrifuge tubes, and 750 μl of the cleared supernatants was used to prepare freezer stocks by mixing with 250 μl of 50% glycerol (12.5% final). Then, 100 μl of this stock solution was used to inoculate the next 10-ml culture. This process was repeated until the supernatant became visibly turbid. Applied forces were calculated using the radial positions of the sampling depths in the centrifuge and the rotational speeds using the formula RCF = 1.12 × radius × (rpm/1,000)^2^. The addition of glycerol to the LB during these selections was incidental because our lab routinely uses LB-glycerol for culturing E. coli to prevent carbon starvation (57). Ingredients for the LB and MOPS-buffered defined media were obtained from Teknova (Hollister, CA).

### Genome sequencing and mutation identification.

Total genomic DNA was purified from each strain, normalized by absorbance, and submitted to the Interdisciplinary Center for Biotechnology Research (University of Florida, Gainesville, FL) for paired-end, 250-nucleotide Illumina sequencing. FASTQ data files were processed in house on a Mint Linux platform to identify mutations. Reads were processed for high quality (trimq = 20) and to remove Illumina adapters and PhiX sequences using the BBduk tool in the BBmap suite (93).

For single nucleotide polymorphism (SNP) and small InDel detection, cleaned reads were mapped to an E. coli MG1655 reference genome (NCBI accession number NC_000913.3) using Bowtie 2 (94). Mapped reads were sorted, and duplicates removed and formatted for variant calling using SAMtools (95). Potential variants were identified using FreeBayes and exported with and without variant calling to spreadsheets for manual inspection (96). The TORMES pipeline was used to compare strains and was used as an alternative read processor before mapping (97). The SNPBac pipeline was used to additionally search for SNPs and short InDels using alternative read processing (SamTools/BCFtools) and mapping (Burrows-Wheeler Aligner MEM algorithm [BWA-MEM]) (95, 98–100). Known variations between the BW30270 experimental strain and the MG1655 reference strain were used to identify variant scores that reflected true sequence changes; as examples, the MG1655 reference sequence has known differences in *gatC* (ACCC to AC) and in *glpR* (CC to CGC) and an inversion in *crl* that are not present in the BW30270 parental strain.

Insertion sequence (IS) element locations were detected using PanISa with reads mapped using BWA-MEM by SNPBac (101). This tool detected new IS*1* element boundaries, which were not detected by the SNP/InDel tools. During this analysis, we discovered that BW30270 has an IS*1* element in *dgcJ*, which is identical in position and orientation to an IS*1* insertion in another published K-12 MG1655 genome sequence (NCBI accession number CP014225.1) (102). We suspect these are the same strain, and the adjacent *cdgI-dgcJ′* deletion recovered in this study is a coincidence.

All variant regions identified by the tools described above were secondarily confirmed by *de novo* assembly of contigs across those regions using PRICE (103). Finally, each mutation region was amplified by PCR and Sanger sequenced to confirm the mutations and to track them during cotransductions.

### Exopolysaccharide purification.

Cultures were transferred to glass tubes and incubated in a boiling water bath for 15 min. After cooling, samples were centrifuged for 10 min at 22,000 RCF, and the supernatants were transferred to clean tubes. Each was then augmented by the addition of CaCl_2_ (0.5 mM final) and MgCl_2_ (2.5 mM final) prior to treatment with 0.25 U/μl of Pierce universal nuclease (Thermo Fisher) for 45 min at room temperature. Proteinase K (New England Biolabs [NEB]) was then added to 0.8 U/ml, and the samples were incubated another 45 min at room temperature prior to another boiling and clearing. Supernatants were then dialyzed twice for 3 h (3,500 molecular weight cutoff [MWCO]) against ∼200 volumes of distilled water (dH_2_O) and overnight against CA buffer (50 mM MES [morpholineethanesulfonic acid], 100 mM NaCl, 0.1 mM EDTA, pH 6.0). Dialysates were centrifuged at 22,000 RCF for 10 min to remove insoluble material, and the supernatants were either stored at 4°C or precipitated with 3 volumes of ethanol prior to resuspension and storage. Methyl-pentose was measured using a colorimetric assay that involved boiling exopolysaccharide samples in sulfuric acid before reacting with cysteine as previously described (4, 5, 42).

### RNA purification.

First, 1 ml of each culture was fixed by mixing with 110 μl of 10% phenol dissolved in ethanol prior to cell harvesting by centrifugation for 30 min at 4°C. After the supernatants were discarded, pellets were resuspended in 200 μl of BE solution (25 mM bis-Tris, 5 mM EDTA, pH 6.5), and then 200 μl of 2% SDS at 50°C was added, and the samples were incubated at 50°C for 1 min to allow thorough lysis. Then, 200 μl of 3 M potassium acetate, pH 4.5, was added and mixed to promote dodecyl sulfate precipitation, and the samples were then cleared by centrifugation for 10 min. Supernatants were decanted into tubes containing 200 μl of 3 M NaCl and 2 μl of 10-mg/ml linear polyacrylamide, mixed, and then extracted one time with a 1:1 phenol-chloroform mixture, pH 4.5 and one time with chloroform, and the RNA was precipitated on ice after mixing with an equal volume of ice-cold isopropanol. Precipitates were harvested by centrifugation for 30 min at 4°C, washed with ice-cold 75% ethanol, followed by 95% ethanol, and air dried. Dried RNA was resuspended in 50 μl of RNA storage buffer (10 mM bis-Tris, 0.1 mM EDTA, pH 6.5).

### Quantitative PCR measurements.

RNA samples were normalized by their absorbance at 260 nM, and 0.1 μg was converted to cDNA using random hexamer priming with the iScript kit (Bio-Rad) using mixtures supplemented with 20 nM of a reverse primer specific for the *rprA* transcript. After synthesis, the cDNA solutions were diluted 20-fold with dH_2_O prior to use as qPCR templates.

Primer pairs were designed to target the 3′ regions of the coding regions of target genes such that the resulting PCR products were ∼110 to 120 bp long. Quantitative PCR was carried out using the SsoFast EvaGreen supermix (Bio-Rad, Hercules, CA) in a CFX96 real-time PCR detection system (Bio-Rad). Fluorescence data were exported and analyzed to obtain template abundance using an online global fitting algorithm at http://www.bioinformatics.org/ucfqpcr (Scilico, LLC) (104, 105). Data averaging and error calculations were performed in Excel (Microsoft, Redmond, WA), statistical analyses and plotting were performed with Prism (GraphPad, San Diego, CA), and figure panels were generated using Photoshop and Illustrator (Adobe, Inc., San Jose, CA).

### CAP attachment to microspheres.

First 1-μm polystyrene beads containing a reactive amine blue dye were purchased from a commercial source (polybeads; Polysciences, Warrington, PA). In pilot experiments, it was discovered that simple adsorption provided comparable adhesion compared to attempted carboxylate/phosphate coupling using 1-ethyl-3-(3-dimethylaminopropyl)carbodiimide hydrochloride. For the reactions shown in Fig. 5, microspheres were diluted to 1/10th their stock concentration in CA buffer, and 200 μl were mixed with 1 ml of CAP dialysates, incubated overnight at room temperature, and then vortexed with an additional 1 ml of CA buffer prior to centrifugation. Subsequent dilutions in CA buffer were used to assess any influence of sample viscosity.

### Disk diffusion assays.

Overnight cultures were diluted 1:5 in fresh medium, and 35 μl was spread onto half of an LB-glycerol plate containing 0.5% sodium dodecyl sulfate (SDS). After liquid absorption, blank 0.25-inch BBL Taxo disks (Becton, Dickinson and Co., Franklin Lakes, NJ) were placed on the inoculated areas, and 12 μl of 250-mM EDTA, pH 7.5, was added to each disk. Plates were incubated overnight and then digitally imaged. For more precise clearance zone measurements, digital images were zoomed, and the clearance zones on the horizontal and vertical axes were measured on-screen. After averaging, the data were rescaled using the known diameter of the disks.

### Biofilm assays.

Biofilms were developed following established protocols for E. coli (106, 107). Overnight cultures of each strain were diluted 1/100 in fresh LB with 0.2% glycerol, and 100 μl aliquots were delivered to wells of a 96-well tissue culture plate. Control wells used for blanking contained sterile growth medium. After 24 h of incubation at 30°C, the plate was shaken to resuspend cells, and the turbidity at 600 nm was recorded. Culture liquid was then aspirated and discarded, and the wells were washed twice with 200 μl dH_2_O prior to staining with 0.1% crystal violet for 15 min. The staining solution was aspirated, the wells were washed 3 times with 200 μl dH_2_O, and then the adherent crystal violet was extracted with 150 μl of 30% acetic acid. After subtracting averaged blank values, the absorbance of crystal violet (550 nm) recovered from each well was divided by that well’s culture turbidity (600 nm). The resulting values from replicate cultures (*n* = 6) were then averaged, and standard deviations were calculated and then normalized as percentages.

## Supplementary Material

Supplemental file 1
