# Supplementary material for: Exopolysaccharide Anchoring Creates an Extreme Resistance to Sedimentation
Source: J Bacteriol. 2021 May 7;203(11):e00023-21. doi: 10.1128/JB.00023-21 (PMC8117528; doi:10.1128/JB.00023-21)

**Supporting Information S1. Strains with sedimentation resistance.** Common *E. coli* research strains partition upon centrifugation such that approximately 1/1000 cells remain in the supernatants. **A)** Comparisons of the sedimentation resistant populations in four lab strains. The total colony-forming units (*CFU*) in 24 h cultures that were grown in LB-glycerol are plotted as well as the CFU of samples taken from the supernatants after centrifugation at 3,000 RCF for 10 min. The error bars represent the standard deviations of four plate counts. The parental "*wild-type*" is a prototrophic MG1655 strain. *MG1655* is a very common *rph-1* strain that exhibits mild auxotrophy for pyrimidines. The fraction of DH5α remaining in the supernatant (super.) was approximately one-tenth that of the other strains. Although much of the data presented in the manuscript employs the use of fixed-angle rotors and microcentrifuge tubes (which might have allowed contamination by cells that were temporarily settled against the outer tube wall), this phenomenon was also observed using swinging bucket rotors and larger volumes. **B)** Separate lineages of *wild-type* cells were serially cultured with selection for sedimentation resistance. After aerobic growth for 24 h in LB-glycerol, samples were centrifuged at 3,300 RCF for 10 min (the selection condition). Shown is a collection of mutants sorted by rank of phenotypic strength. From this collection, strains exhibiting a *weak*, *moderate*, or *strong* resistance phenotype were selected for detailed study. Samples 4, 5, and 6 are from the same lineage at different generations and have the same deletion in *lpp*.

Kessler Fig. S1

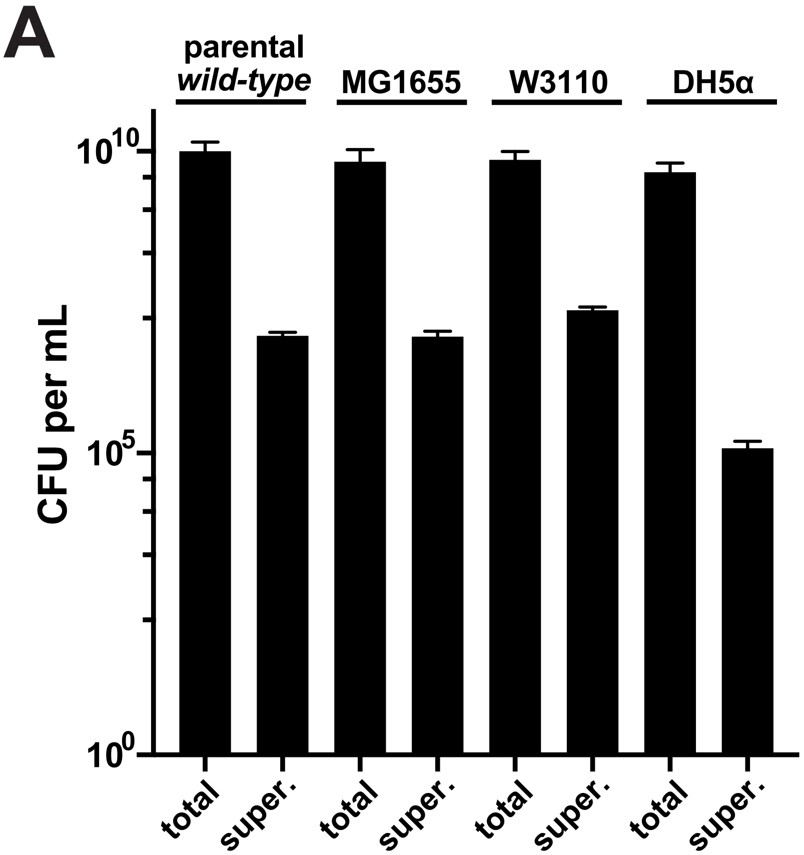


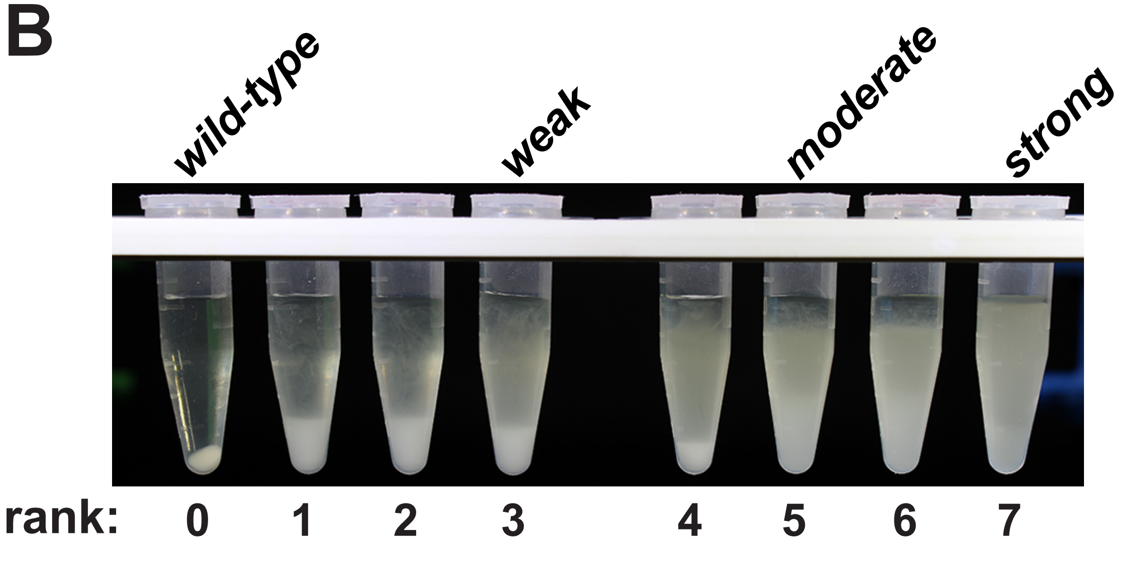

**Supporting Information S2. Details of the *IS1* mutations.** The parental strain has five established *IS1* elements. The *moderate* and *strong* mutants experienced *IS1* mobile element migrations and the *strong* mutant also acquired a deletion adjacent to a preexisting *IS1* insertion. **A)** Schematics of the *IS1* alterations in the *moderate* mutant with new/deleted DNA highlighted in pink. The ORF of *yebE* was disrupted as well as the non-coding region between *yjbE* and *yjbF*. The *IS1* elements are drawn with their orientations relative to the promoter that drives expression of *insA* and *insB*. **B)** *IS1* alterations in the *strong* mutant involve a deletion of *cdgI*, an insertion in the *cps* promoter (between the JUMP element and the beginning of the *wza* ORF), and an insertion in *yjbF*.

**Kessler Fig. S2**

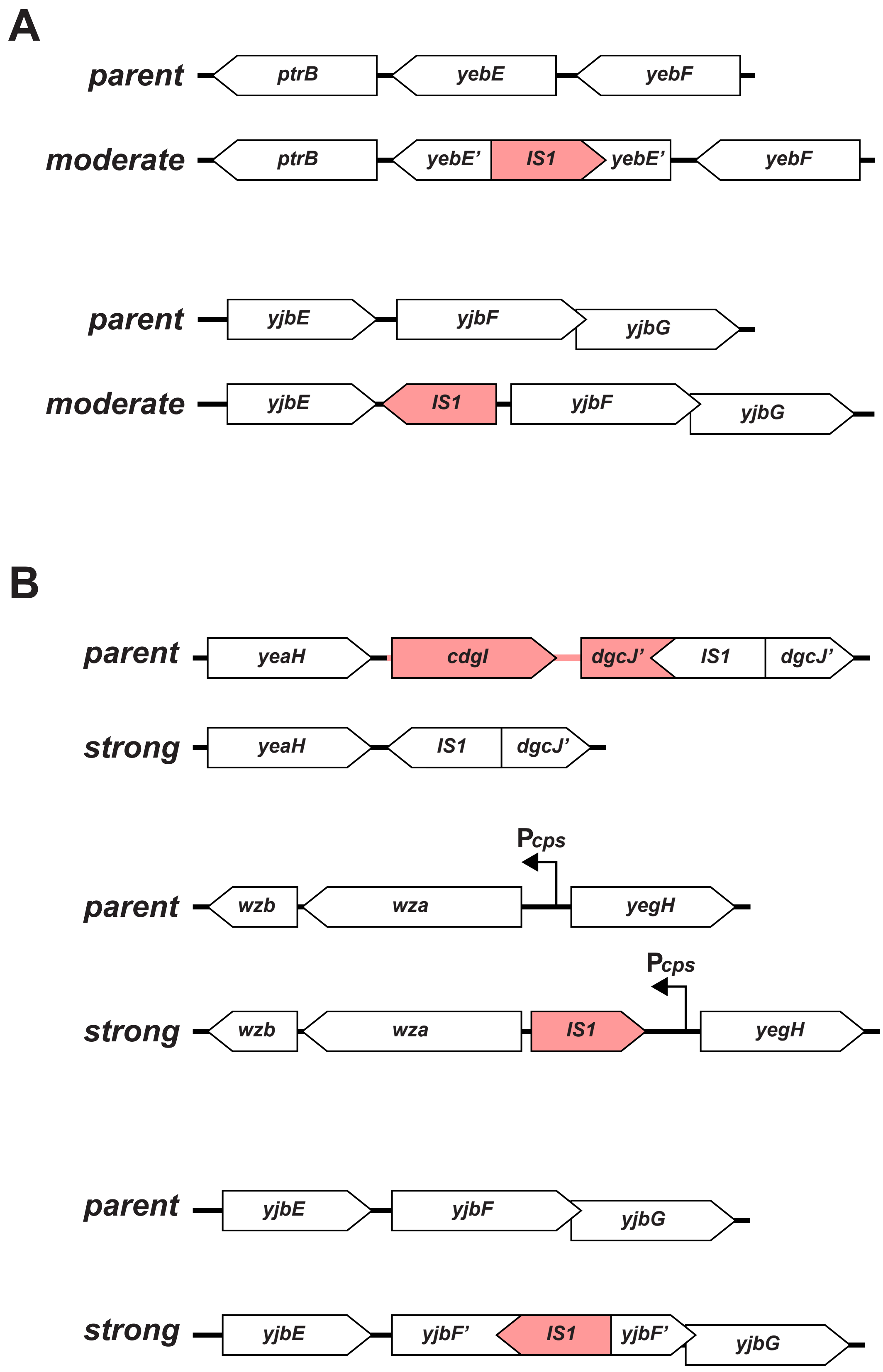

**Figure S3. Transcript levels of other Rcs targets.** Real-time, quantitative PCR of cDNA was used to measure transcript levels in samples extracted from exponential and 24 h stationary cultures. **A)** Exponential phase cultures of each after centrifugation for RNA harvesting. The pellet of the *moderate* mutant was less compact than the others, consistent with the level of activation of the Rcs system at this growth stage. **B)** The levels of *rcsA* mRNA. **C)** The levels of *yjbG* mRNA.

Kessler Fig. S3

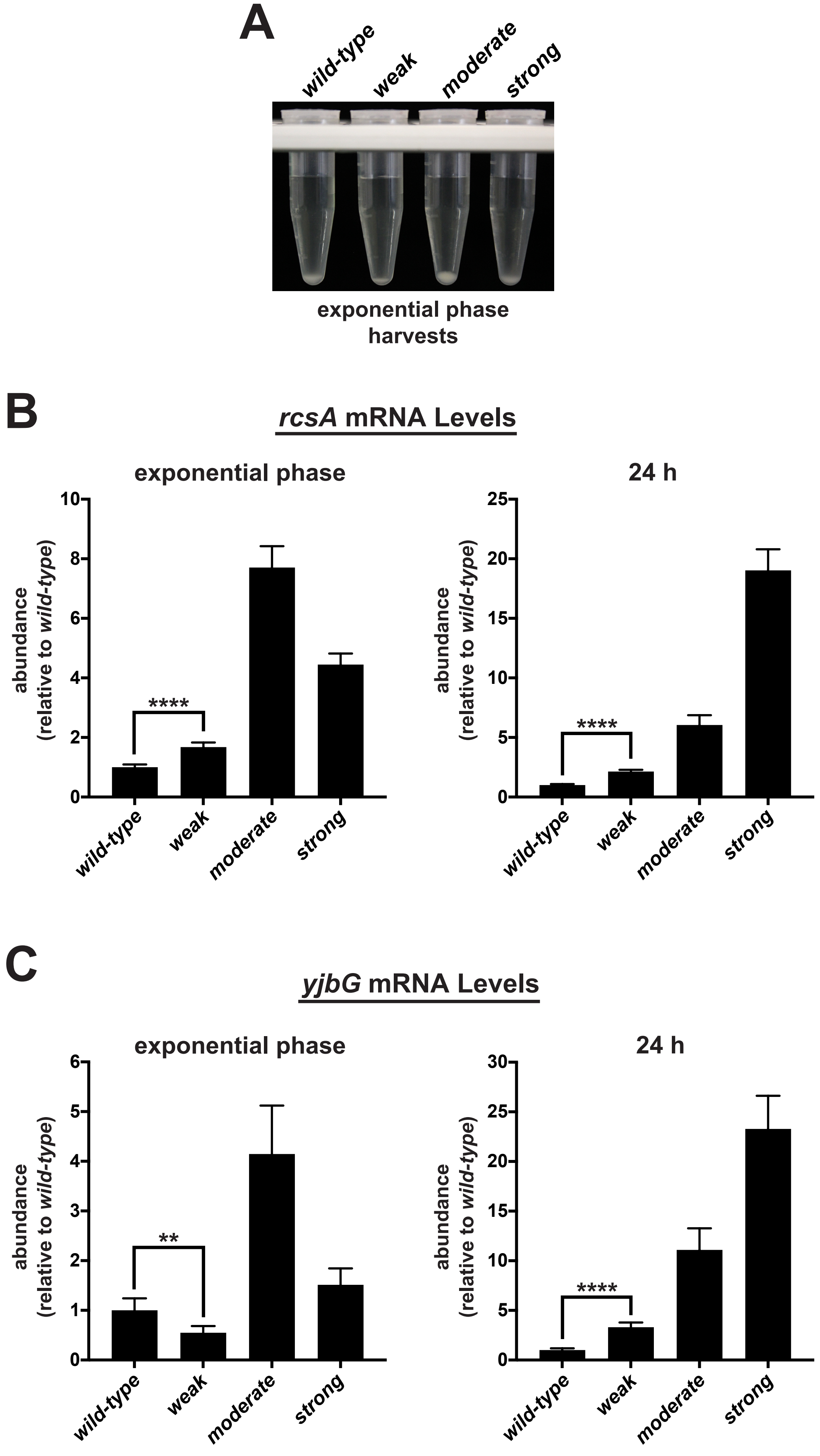

**Supporting Information S4. Centrifuging dilutions and *wild-type* mixing.** The mutants were evaluated for intrinsic resistances to sedimentation. **A)** Aliquots of cultures of each strain were serially diluted and plated so the CFU/mL of the original cultures could be established (*centrifuged:* -). Those dilution tubes were subsequently centrifuged at 3,300 RCF for 10 min and samples of the supernatants were also plated to establish "residual" CFUs from the cells remaining in suspension (*centrifuged:* +). Mutant samples that had been diluted $10^6$ or $10^7$ yielded colonies similar in number to the original cultures, indicating that they had not substantially migrated. Error bars are standard deviations from three experimental replicates. **B)** A culture of *wild-type* cells was mixed 1:1 with cultures of mutant cells and centrifuged at 3,300 RCF for 1 min (to limit the sedimentation of the *weak* mutant). The *wild-type* cells formed a discernable dense pellet in proportion to their abundance, indicating that the mutant cells had not retarded their migration.

Kessler Fig. S4

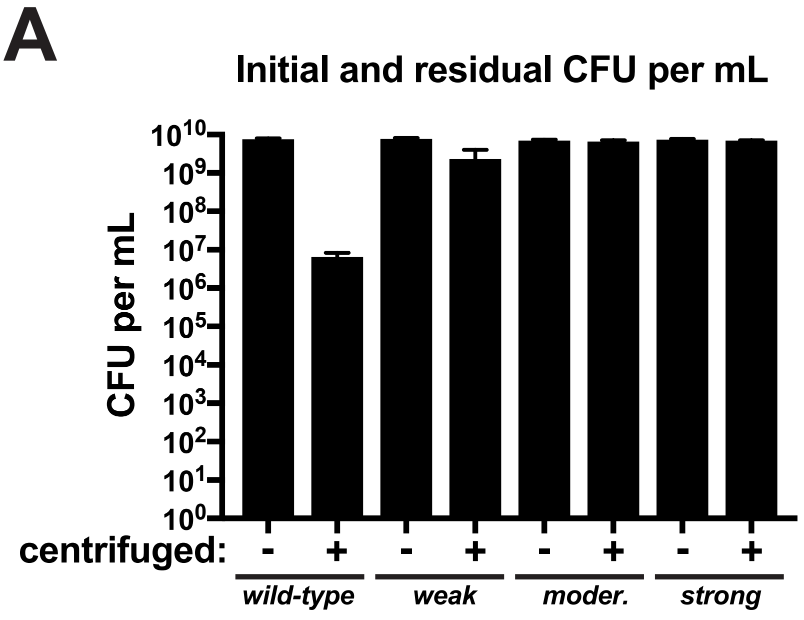


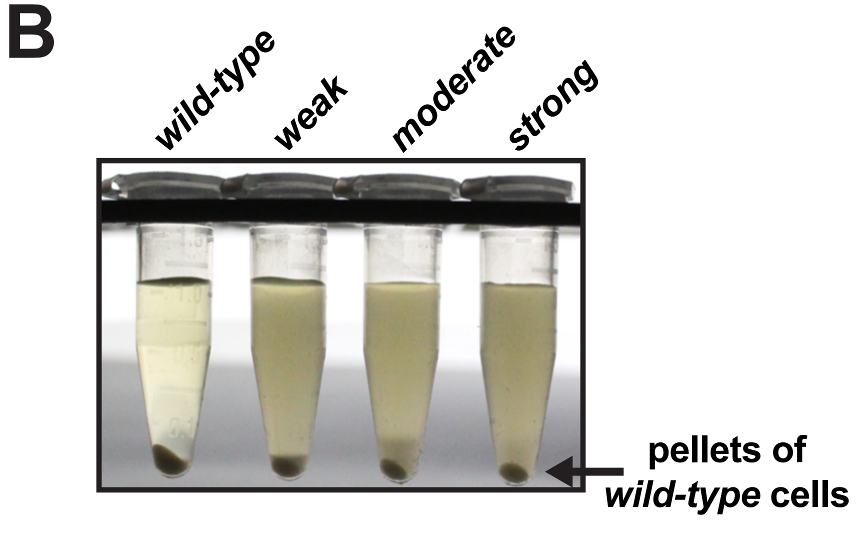

**Figure S5. Growth rates and stress challenges.** The mutants were evaluated for fitness relative to the *wild-type* parental strain. **A)** Cultures of each were grown aerobically with shaking in non-supplemented or in 0.2% glycerol-supplemented LB at 30 or 37 °C and the culture turbidities recorded every 5 min. *Left panel*: representative $\log_2$ plots of the turbidity data for cultures grown in non-supplemented LB at 30 °C. The data were shifted in increments of -2 units to allow easier visual comparisons. Derivatives of each data set were separately calculated and plotted to identify the fastest exponential growth regions in each culture, which corresponded to the ~30 min window preceding an $Abs_{600}$ of 0.05 (highlighted with colors). *Right panel*: plots of the $\log_2$ regions of those data that were fitted using linear regression to obtain doubling times, which were reciprocals of the slopes. **B)** The exponential growth rates from three separate experiments were averaged and plotted with their standard deviations. The only significant growth rate differences were between the *wild-type* cultures and the *moderate* and *strong* mutants in non-supplemented LB at 30 °C. **C)** Doubling times of cultures grown at 30 °C in LB-glycerol prepared with 0.25, 0.5, 1, or 2 % NaCl. The salt stresses became substantial at 2 % NaCl, but the mutants grew as well as *wild-typ*e. **D)** Resistances to antibiotics that target cell wall synthesis in the periplasm (ampicillin) or gyrase in the cytosol (ciprofloxacin) were measured in 96-well plate culture assays. The drug concentrations at the MIC boundaries are indicated. **E)** Persistence was evaluated by enumerating the surviving populations after treatment with inhibitory doses of ampicillin or ciprofloxacin. *Left panel*, overnight cultures were diluted 1:100 in fresh LB-glycerol containing either 100 ug/mL ampicillin (*amp*) or 1 ug/mL ciprofloxacin (*cipro*). After 4.5 hours of incubation at 30 °C, samples were serially diluted for plate counting.

*Right panel*, cultures prepared similarly to those in the left panel, except the bacteria were allowed to first enter exponential phase by pre-incubating them for 2 h at 30 °C, treated with antibiotic, and then cultured for an additional 3.5 h prior to counting. The error bars are the standard deviations from three separate cultures of each. Not shown: the surviving *wild-type* cells (persistent) were not resistant to sedimentation when their serial dilutions were centrifuged and plated (CFU dropped below the limit of detection). Therefore, the sedimentation-resistant populations are not in the persistent state.

**Kessler Fig. S5**

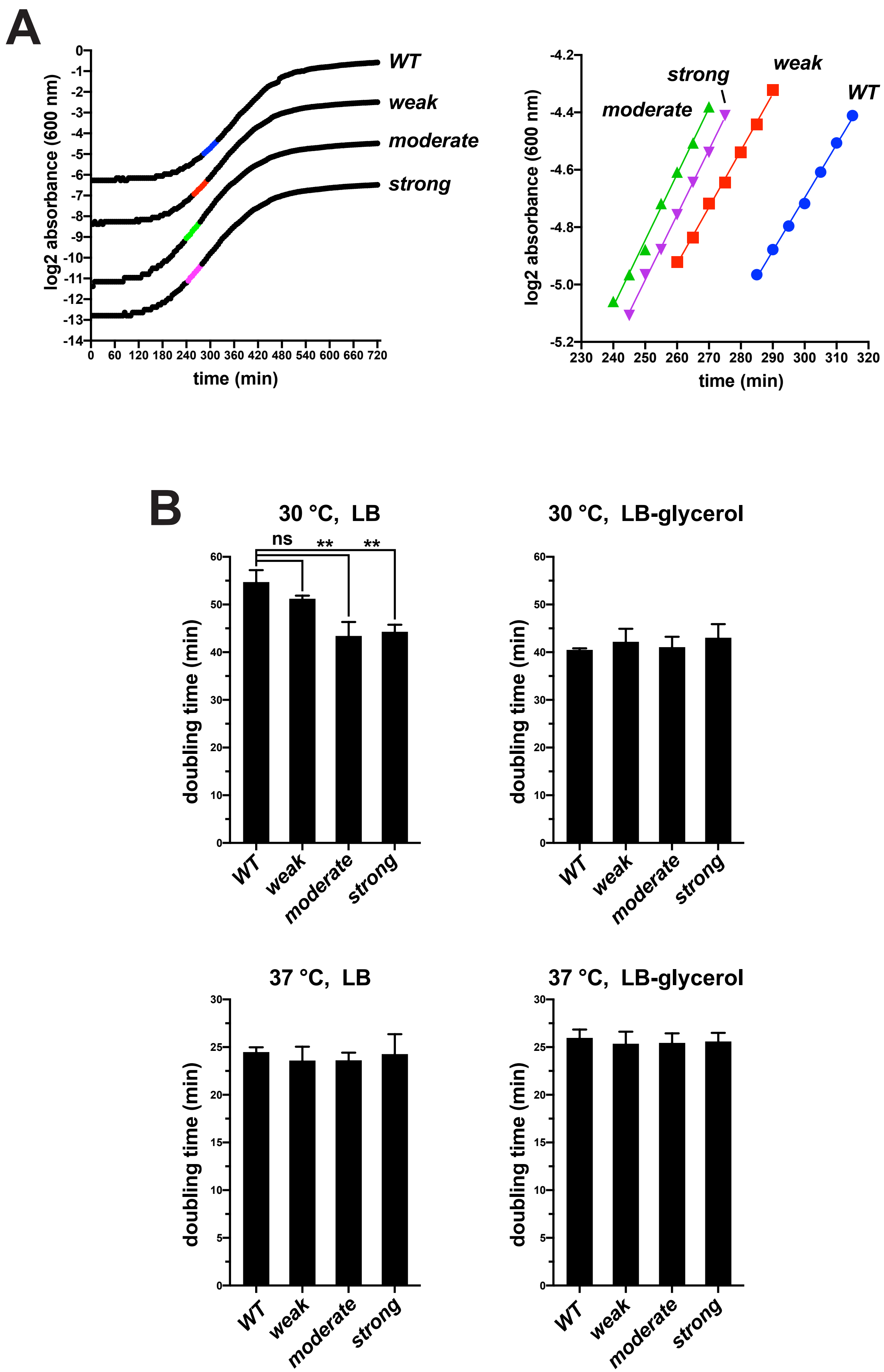

# Kessler Fig. S5_continued

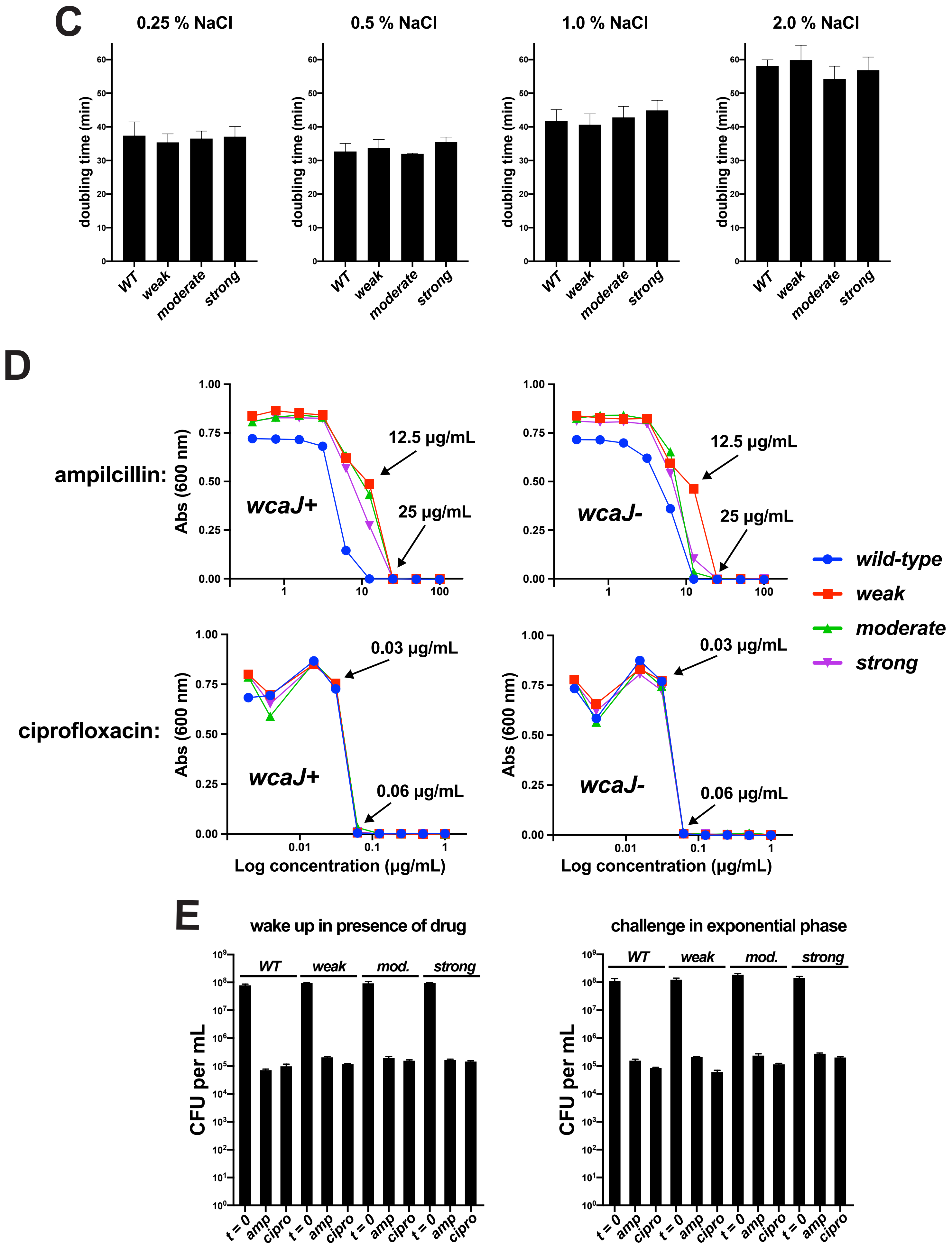

**Supporting Information S6. Sedimentation resistance under different conditions.**

**A)** Cultures were grown in defined, rich medium (containing EZ supplement, lacking ACGU supplement). Although all phenotypes were less pronounced than when grown in LB, the notable influence of glycerol on the *weak* mutant's phenotype remained. **B)** Cultures were grown in MOPS-buffered minimal media (lacking the "EZ" supplement that provides amino acids, thiamine, and benzoic acids and lacking the purine/pyrimidine ACGU supplement) for 24 h at 30 °C with aeration prior to centrifugation. With 0.2% glycerol or no additional carbon source, the growth of all cultures was substantially reduced or undetectable. With glucose present, the *moderate* mutant grew to a higher cell density and also exhibited sedimentation resistance. **C)** Left panel, the parental, *wild-type* strain was grown in LB in the presence and absence of a 0.2% glycerol supplement to evaluate sedimentation resistance. Total cultures and samples of supernatants were serially diluted and plated to establish CFU. The sedimentation resistance was not dependent on the added glycerol. Right panel, the same experiment, but with a *ΔwcaJ::kan* derivative of the *wild-type* strain.

Kessler Fig. S6

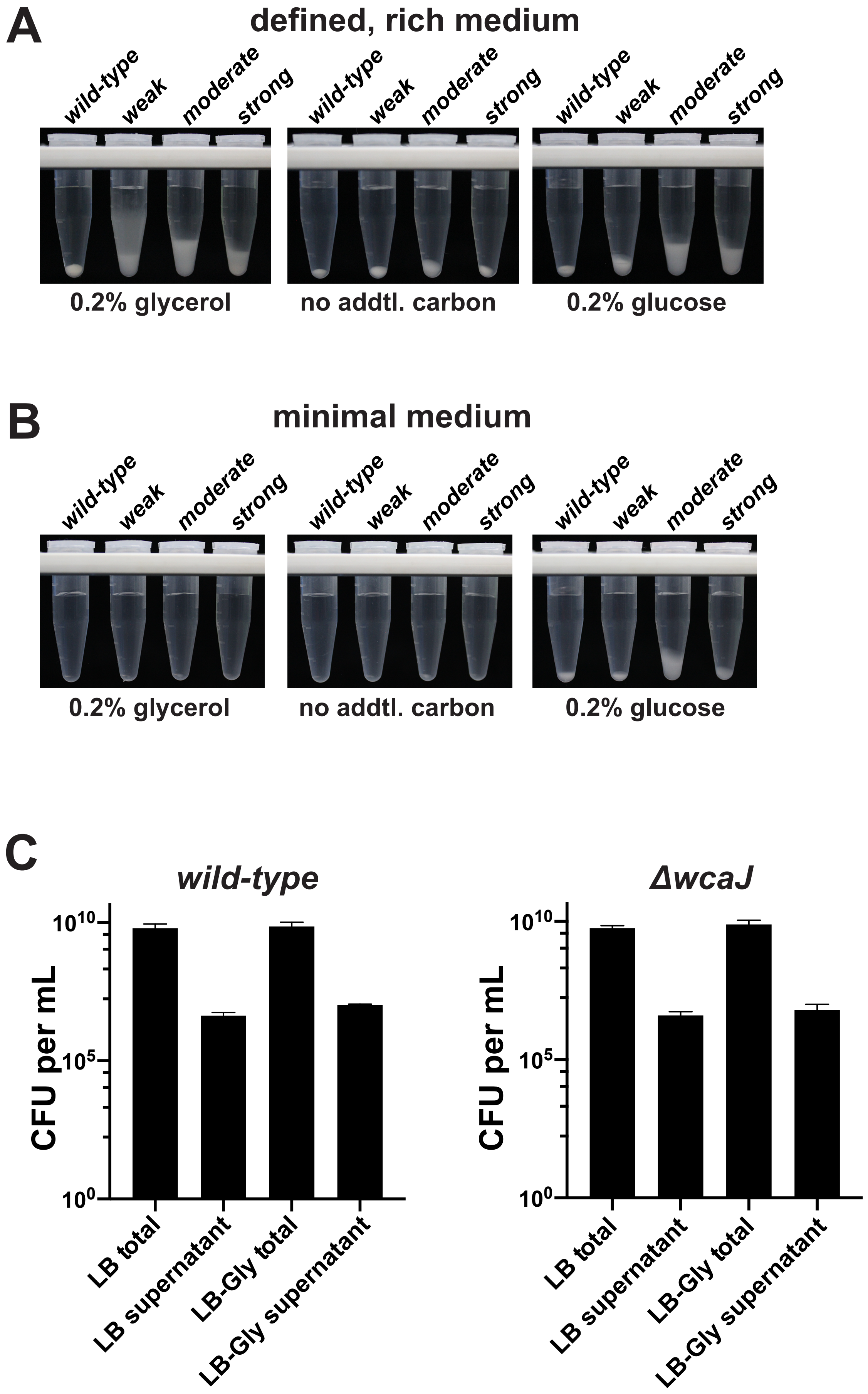

Supplement: Supplemental file 1 [file JB.00023-21-s0001.pdf]
